# Highly Absorbent Ultrafast Self‐Gelling Starch Microparticles for Robust Wet‐Tissue Adhesion and Instant Hemostasis

**DOI:** 10.1002/advs.202501857

**Published:** 2025-03-24

**Authors:** Soohwan An, Jihoon Jeon, Seung Yeop Han, Mi Jeong Lee, Tae‐Gyeong Oh, Eun Je Jeon, Dong Jin Joo, Seung‐Woo Cho

**Affiliations:** ^1^ Department of Biotechnology Yonsei University Seoul 03722 Republic of Korea; ^2^ Department of Biomaterials Science and Engineering Yonsei University Seoul 03722 Republic of Korea; ^3^ CellArtgen Inc. Seoul 03722 Republic of Korea; ^4^ Department of Surgery Severance Hospital Yonsei University College of Medicine Seoul 03722 Republic of Korea; ^5^ Center for Nanomedicine Institute for Basic Science (IBS) Seoul 03722 Republic of Korea

**Keywords:** absorbent & adhesive hydrogel, hemostasis, microparticle, starch, ultrafast self‐gelation

## Abstract

Rapid and effective hemostasis of mass bleeding from irregularly shaped wounds remains a critical clinical challenge. Herein, a highly absorbent and self‐gelling microparticle (MP) is reported based on modified starch as a hemostatic material with robust wet tissue adhesiveness. The aldehyde‐ and catechol‐modified starch (ACS) is synthesized via partial oxidation of starch for the reduction of dense intermolecular interactions within starch, thereby significantly enhancing its interaction with water molecules. Moreover, the conjugated catechol group strengthens the affinity to various biomolecules. The ACS microparticle (ACS‐MP) prepared by calcium intercalation into the polysaccharide chains and subsequent freeze‐milling rapidly converts to a stable hydrogel within seconds upon hydration, exhibiting superior water absorption capacity and tissue adhesion. The ACS‐MP demonstrates excellent in vivo biocompatibility in local and systemic administration. The application of ACS‐MP to bleeding wounds enables rapid blood absorption and accumulation of blood components and coagulation factors. The ACS‐MP can fill irregularly shaped wounds, forming a tissue‐adhesive hydrogel in situ, thereby creating a physical barrier for non‐compressible hemostasis. The superior hemostatic performance of ACS‐MP against massive hemorrhage from liver injuries in mice and pigs is confirmed. The ACS‐MP will be a promising hemostat for effectively controlling mass bleeding in various tissues in clinical settings.

## Graphical abstract

The aldehyde‐ and catechol‐modified starch (ACS) microparticle (MP) is developed by chemical modifications and freeze‐milling after calcium intercalation for highly absorbent and tissue‐adhesive properties. The ACS‐MP can be self‐gelled upon contact with biological fluids and blood on tissue surfaces, rapidly absorbing them and achieving robust wet‐tissue adhesion. These unique characteristics synergistically facilitate the superior hemostatic performance of ACS‐MP in a massive bleeding liver.

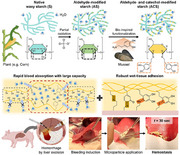

## 1 Introduction

During severe traumatic injuries or surgical procedures, the endogenous hemostasis mechanism in our body is not enough to achieve effective bleeding control in a short time.^[^ ^1^ ^]^ The uncontrolled, excessive bleeding leads to severe morbidities and even death.^[^ ^2^ ^]^ Thus, various types of hemostatic materials have been actively developed over the past decades, especially for addressing acute and massive bleeding. Ideal hemostatic materials should have the following requirements: rapid uptake of large amounts of blood to accumulate the blood components and coagulation factors, fast and tight physical blocking with adequate tissue‐adhesiveness on the irregularly shaped wounds, excellent biocompatibility, and inherent biodegradability.^[^ ^3^ ^]^

Starch is a natural polymer that has been used as a main energy source and can be biodegraded in many animals, including humans, as a Generally Recognized as Safe (GRAS) ingredient,^[^ ^4^ ^]^ hence it has been widely used in industrial and biomedical applications. Furthermore, starch can be modified to have highly absorbent properties for fabricating hydrogels and super‐absorbents, although native starch has poor water solubility due to the highly branched structure constructed by α‐1,6 glycosidic bonds in amylopectin.^[^ ^5^ ^]^ Additionally, due to the abundant hydroxyl groups that can form hydrogen bonding, starch can exhibit inherent adhesiveness to various surfaces, which has been used as an industrial adhesive like wheat paste for a long time.^[^ ^6^ ^]^ Considering these unique and beneficial characteristics, starch will likely meet all the requirements for ideal hemostatic materials.

Despite these advantages of starch, incorporating water molecules into the starch polymers to fabricate water‐interactive biomaterials like hydrogels for improved bioavailability usually requires high temperatures or harsh pH conditions for gelatinization.^[^ ^7^ ^]^ These requirements may significantly limit starch's practical and clinical application as an instantly used or in situ‐operating biomaterials. Furthermore, the origin and mechanism of its inherent adhesiveness are highly dependent on hydrogen bonding and usually require additional hardening by drying it for solid adhesion,^[^ ^8^ ^]^ which may interfere with its sufficient adhesion to the wet tissue surfaces covered with biological fluid and blood in the body. Thus, these drawbacks have resulted in the limited use and performance of starch in biomedical applications despite its excellent functionality and safety.

**Figure 1:**
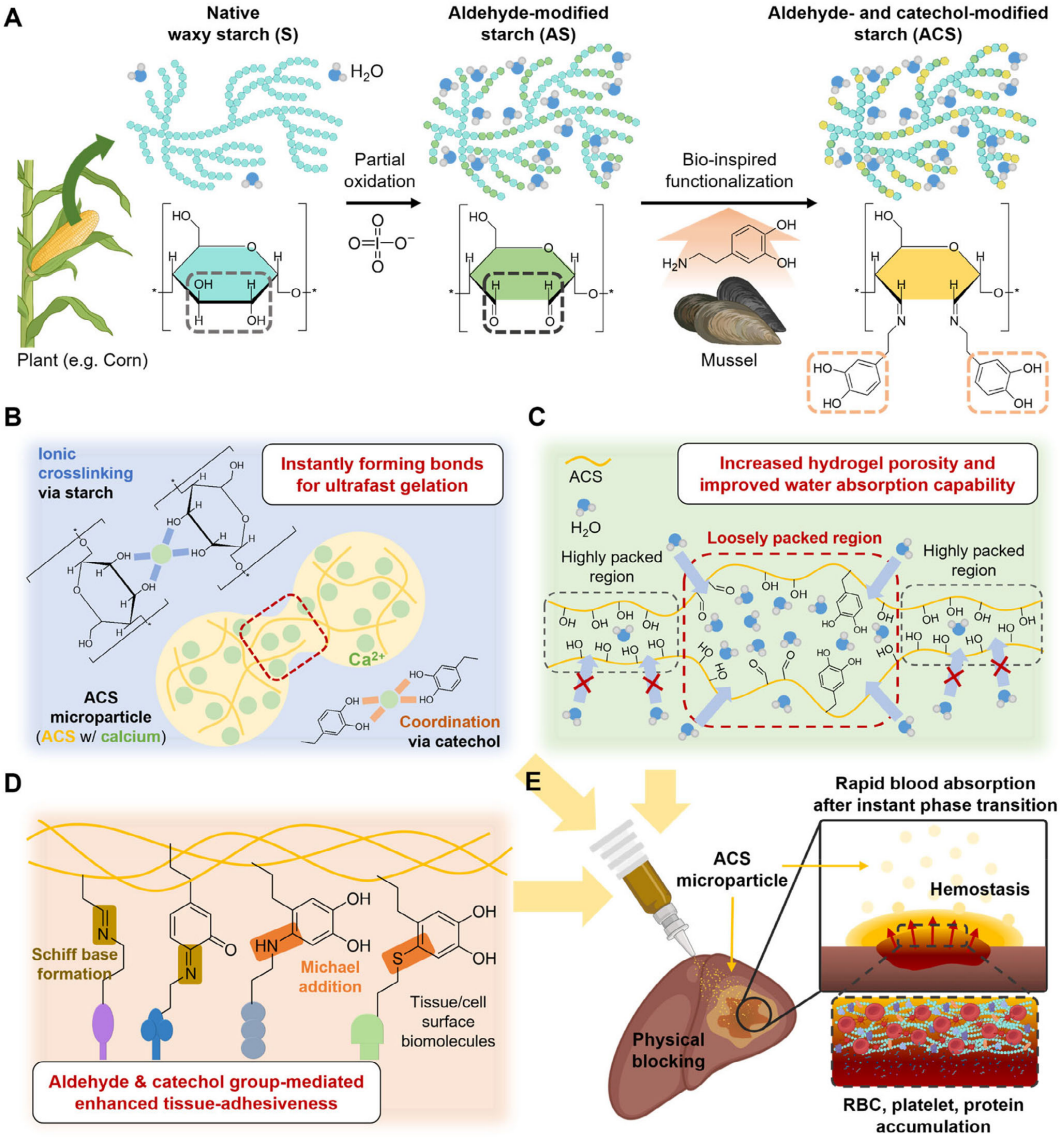
Synthesis of ACS polymer and characteristics of ACS microparticle (MP). A) Schematic illustration of the synthesis procedures of the ACS polymer and water accessibility of the starch polymer in each stage. B) Calcium‐mediated instantly forming bonds among the ACS polymers in the ACS‐MP. C) Enhanced water accessibility of the ACS polymer and improved water absorption capability driven by chemical modifications. D) Chemical bonding of aldehyde group and catechol group in the ACS polymer with several functional groups in biomolecules for robust bio‐adhesive properties of the ACS‐MP. E) Plausible mechanisms of synergistically enhanced hemostatic capability of the ACS‐MP.

Herein, to address these issues above, corn‐derived starch containing essentially pure amylopectin and only trace amounts of amylose was partially oxidized by using sodium periodate that can oxidize vicinal diol to aldehyde groups (**Figure** **1**A). As a result, the number of hydroxyl groups and their repetitive arrangement decreased, reducing the dense intermolecular interactions within the polysaccharide chains.^[^ ^9^ ^]^ This change makes the aldehyde‐modified starch (AS) more easily accessible to water molecules, forming more hydrogen bonds with them. Additionally, dopamine, a natural catecholamine, was easily conjugated to the aldehyde groups in the AS through a Schiff base linkage. This process introduced the catechol group, a crucial functional element for the underwater adhesion observed in mussels^[^ ^10^ ^]^ to the AS, resulting in the formation of aldehyde‐ and catechol‐modified starch (ACS). The ACS polymer was pre‐processed in several steps to intercalate calcium into the ACS polysaccharide chains. This enabled the instant formation of calcium ion‐mediated ionic crosslinking and coordination for ultrafast gelation in wet conditions (Figure 1B). Furthermore, the physical and steric hindrance resulting from the two‐step chemical modifications of starch may interrupt hydrogen bonding between the polymers and reduce the rigidity of the polymers, thereby increasing the water accessibility of the ACS.^[^ ^11^ ^]^ This would contribute to the enhanced water absorption capability of the ACS‐based hydrogel due to increased porosity of the hydrogel (Figure 1C). Especially, the introduced aldehyde and catechol groups can interact in various ways, including covalent bonding via Schiff base and Michael addition, with diverse functional groups in biomolecules,^[^ ^12^ ^]^ contributing to enhanced tissue‐adhesiveness (Figure 1D). The ACS with calcium ion can be processed to manufacture a microparticle (MP) form, which not only facilitates effective and minimally invasive access to irregular defects but also increases the surface area to maximize water absorption capability and adhesiveness compared to bulk hydrogels.^[^ ^13^ ^]^ As a result, upon application of the ACS‐microparticles (ACS‐MPs) to the bleeding site, it can be quickly transformed into the adhesive hydrogel onto the defective tissue to physically block the bleeding, simultaneously absorbing the blood and accumulating blood components for effective hemostasis (Figure 1E). To ascertain the superior hemostatic performance of ACS‐MPs, a liver hemorrhage model in both mice and pigs was used in this study, where the ACS‐MPs exhibited potential as a highly biocompatible and functional hemostatic hydrogel.

## 2 Results and Discussion

### 2.1 Production and Characterization of Starch‐Based MPs

**Figure 2:**
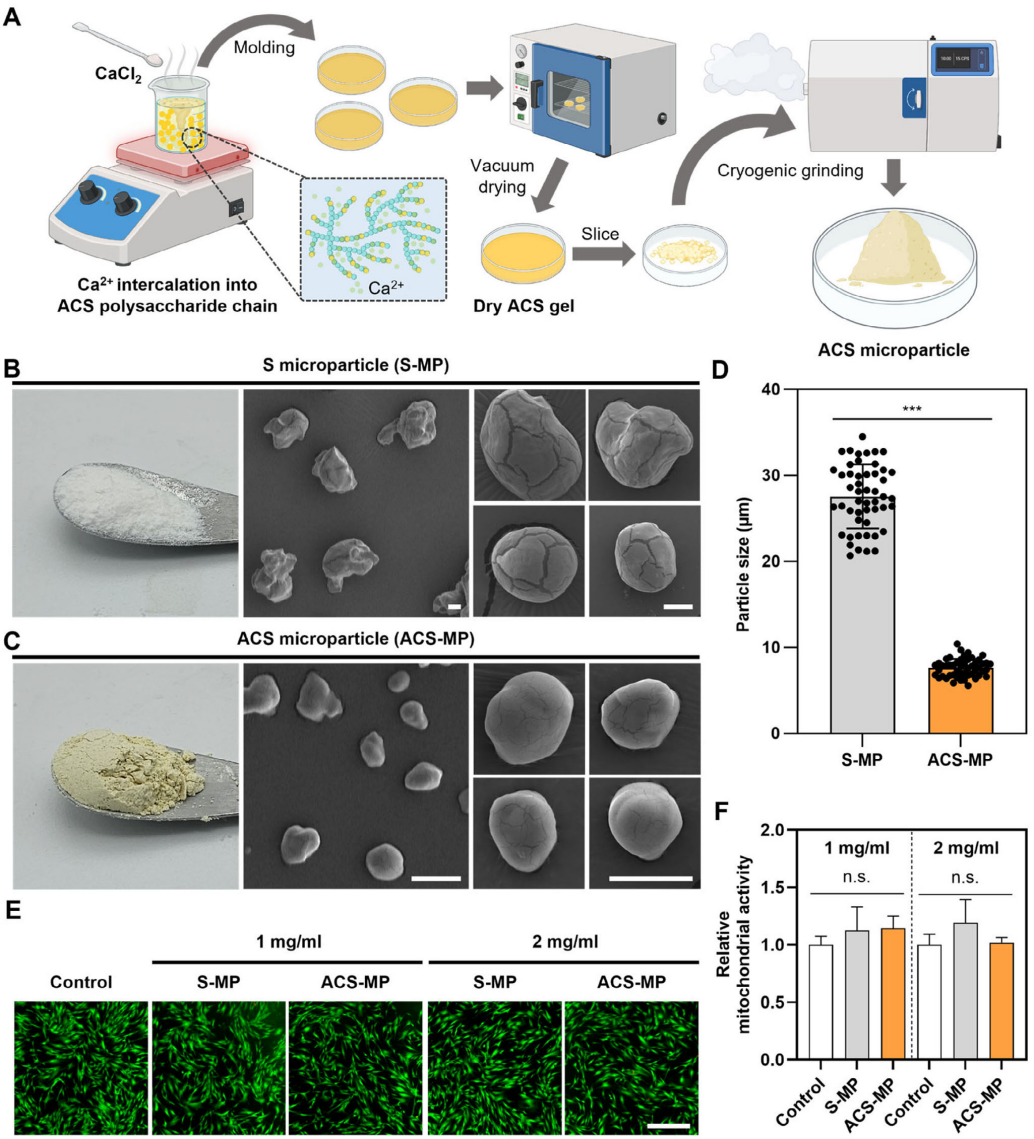
Preparation and characterization of ACS‐MP. A) Schematic illustration of fabrication processes of the ACS‐MP. Photographs and SEM images of B) S microparticle (S‐MP) and C) ACS microparticle (ACS‐MP) (scale bars = 10 µm). D) Quantification of the particle size of the S‐MP and ACS‐MP (*n* = 50; ^***^ *p* < 0.001). In vitro biocompatibility of each MP as confirmed by E) Live/Dead staining assay and F) MTT assay (*n* = 5) of human neonatal dermal fibroblasts 24 h after culture in each MP‐conditioned medium.

The percentage of aldehyde‐modified glucose in the AS was ≈40%. The degree of substitution of catechol group in the ACS polymer was ≈11% (Table S1, Supporting Information). Both starch‐based MPs can be easily produced by three simple steps (**Figure** **2**A). Starch or ACS was dispersed in CaCl_2_ solution, and the solution was heated with vigorous agitation to break the starch intermolecular interactions partially and temporarily for intercalating calcium ions between the polysaccharide chains. Then, the mixture was molded and vacuum‐dried for stabilization to prepare dry starch gels, which were cryogenically ground, resulting in the production of the starch‐based MPs (Figure S1, Supporting Information). The MP made of native starch (S microparticle; S‐MP) showed irregular and agglomerated appearances (Figure 2B). In contrast, the MP made of ACS (ACS microparticle; ACS‐MP) showed relatively uniform and globular shapes (Figure 2C). The average particle size of ACS‐MPs was ≈7.6 µm in diameter, which is ≈3.6 times smaller than that of S‐MPs (Figure 2D). Also, the particle size distribution of ACS‐MPs was much narrower than that of S‐MPs. This was likely due to the mechanically stiffer and more brittle dry ACS gels that underwent more fracture and abrasion during physical grinding compared to dry S gels (Figure S2, Supporting Information).^[^ ^14^ ^]^ The smaller size of ACS‐MPs contributes to the increased surface areas, thereby enhancing the performance of the MPs for contacting and interacting with other substances, such as biological fluids and biomolecules.

Since the main ingredients of both starch‐based MPs are starch with calcium, the MPs are highly biocompatible. The in vitro cytocompatibility of each MP‐conditioned medium was comparable to that of the control medium in culturing fibroblasts for 24 h, regardless of the concentrations of MPs in each conditioned medium (Figure 2E). Quantitatively, the viability of fibroblasts cultured in both MP‐conditioned media showed no statistically significant differences compared to the control group, indicating that both starch‐based MPs exhibited excellent cytocompatibility (Figure 2F).

### 2.2 Wetting‐Induced Ultrafast Self‐Gelation of ACS‐Based MPs

**Figure 3:**
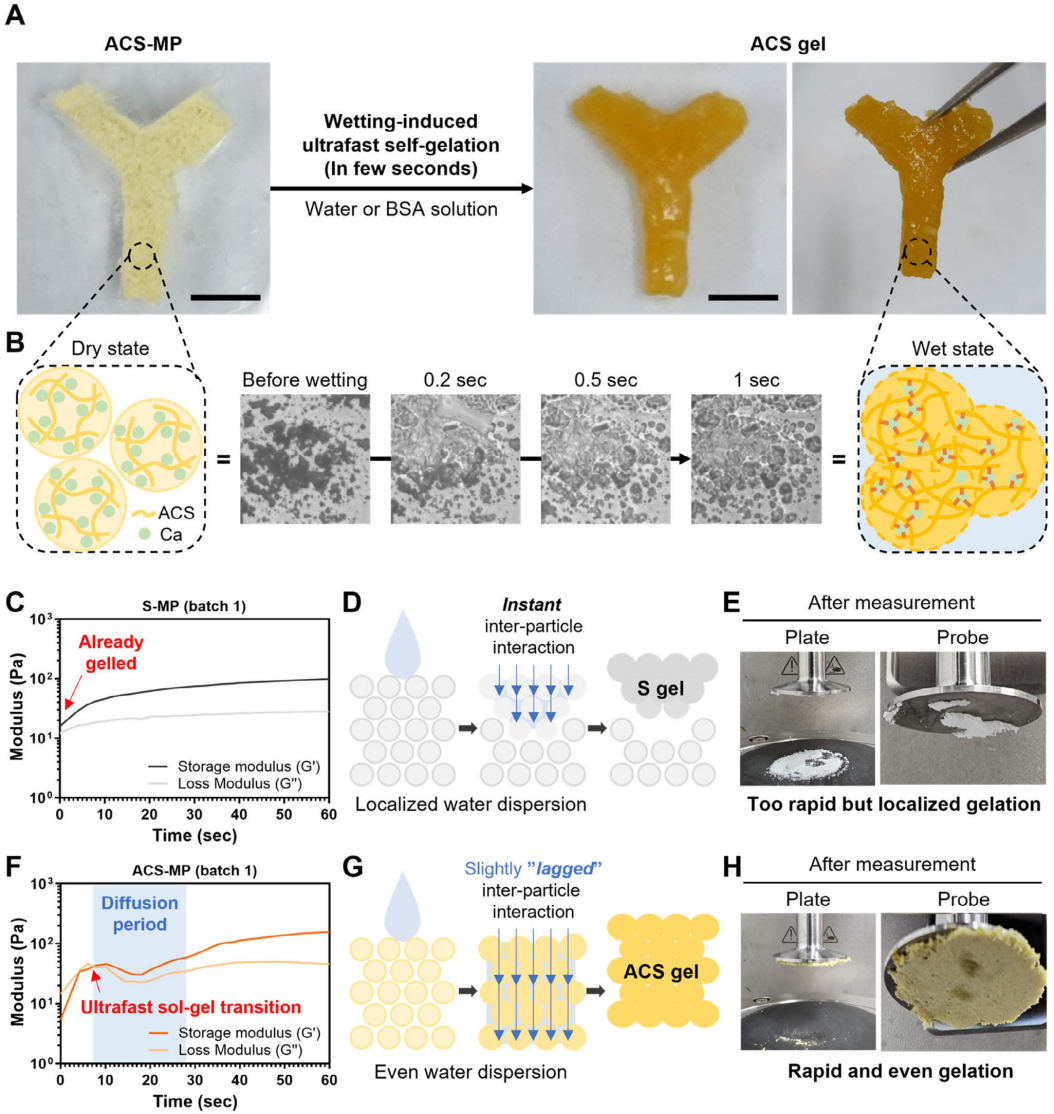
Gelation properties and kinetics of ACS‐MP. A) Photographs of the ACS‐MP in a Y‐shaped mold (left) and the ACS gel after wetting‐induced ultrafast self‐gelation by treating with BSA solution (right) (scale bars = 5 mm). B) Schematic illustration and microscopic images of ACS‐MPs before and after wetting. C) Gelation kinetics of the S‐MP upon absorbing BSA solution as investigated by rheological analysis. D) Schematic illustration of a situation in which S‐MPs come in contact with an aqueous solution and form instant inter‐particle interactions. E) Photograph of the S‐MPs on the plate and probe of a rheometer after measurement. F) Gelation kinetics of the ACS‐MP upon absorbing BSA solution as investigated by rheological analysis. G) Schematic illustration of a situation in which ACS‐MPs come in contact with an aqueous solution and form slightly lagged inter‐particle interactions. H) Photograph of the ACS‐MPs on the plate and probe of a rheometer after measurement.

The ACS‐based MPs were prepared in a lyophilized form, where the interaction between the individual particles was entirely restricted (**Figure** **3**A). Upon exposure to aqueous solutions, ACS‐MPs absorb the solution and swell rapidly. When the swollen particles come into contact with neighboring particles, the starch polysaccharides and calcium ions in the wet particles become locally mobile, allowing them to interact with other polysaccharide chains and calcium ions in the neighboring particles. (Figure 3B; Video S1, Supporting Information). Simultaneously, calcium ion‐mediated bonds between starch polysaccharide chains in the swollen particles are instantly formed,^[^ ^15^ ^]^ which leads to the formation of interpolymer networks within and between the particles within a few seconds, enabling the ultrafast self‐gelation of the ACS‐MPs into a stable hydrogel. In X‐ray photoelectron spectroscopy (XPS) analysis, the Ca2p spectrum of the resultant ACS hydrogel consists of four peaks including the peaks corresponding to calcium bonded to oxygen in the O─Ca─O bondings at 346.8 eV (2p_3/2_) and 350.2 eV (2p_1/2_) (Figure S3, Supporting Information), which mainly contribute to stable gelation of the Ca‐starch‐based hydrogels.^[^ ^16^ ^]^ These phenomena could be similarly observed in the case of S‐MP, and the storage moduli of hydrogels made of both MPs were consistently higher than the loss moduli in the frequency range of measurement (Figure S4A,B, Supporting Information). These results indicate that the hydrogels were stably formed via self‐gelling mechanisms and behaved as viscoelastic materials, although their mechanical properties may decrease as the hydrogels absorb a larger amount of solution and are more swollen (Figure S4C, Supporting Information). Furthermore, the modulus of self‐gelled starch hydrogels (≈1–20 kPa) can cover that of various tissues, such as liver (≈5 kPa), stomach (≈1.9 kPa), lung (1–5 kPa), brain (0.1–3 kPa), skin (0.2–2 kPa), muscle (10–18 kPa), and adipose tissue (2–5 kPa),^[^ ^17^ ^]^ allowing for adequate mechanical matching with the tissues having irregular shapes.^[^ ^18^ ^]^

When investigating the rheological properties of both starch‐based MPs during wetting‐mediated self‐gelation in a time‐sweep mode, each MP showed distinct gelation kinetics. In the case of S‐MPs, the storage modulus was higher than the loss modulus immediately after wetting them, indicating that they were gelled right away (Figure 3C; Figure S5A, Supporting Information). However, water was not well dispersed among the S‐MPs and was absorbed by only some particles, forming instant inter‐particle interactions in the localized region (Figure 3D). Despite the extremely rapid gelation of S‐MPs, only part of the particles seemed to participate in the gelation and eventually fail to form a uniform hydrogel (Figure 3E). Interestingly, the gelation of ACS‐MPs showed unique rheological changes. The storage modulus crossed over the loss modulus within very few seconds (average time: ≈3∼4 s) upon exposure to wet conditions, indicating gelation initiation, but the storage and loss moduli were maintained at similar levels for ≈20 s even after crossover (Figure 3F; Figure S5B, Supporting Information). The intermolecular interactions within the ACS would be physically and sterically hindered owing to the introduction of aldehyde and catechol groups via the two‐step chemical modifications, which leads to higher accessibility and affinity of ACS to water molecules. As a result, water can diffuse much more easily into the starch polymer, and the inter‐particle interactions between ACS‐MPs would be slightly lagged within the “diffusion period,” which facilitates more even water dispersion between the ACS‐MPs, enabling their rapid and even gelation (Figure 3G,H).

### 2.3 Improved Water Absorption Properties of ACS Gel

**Figure 4:**
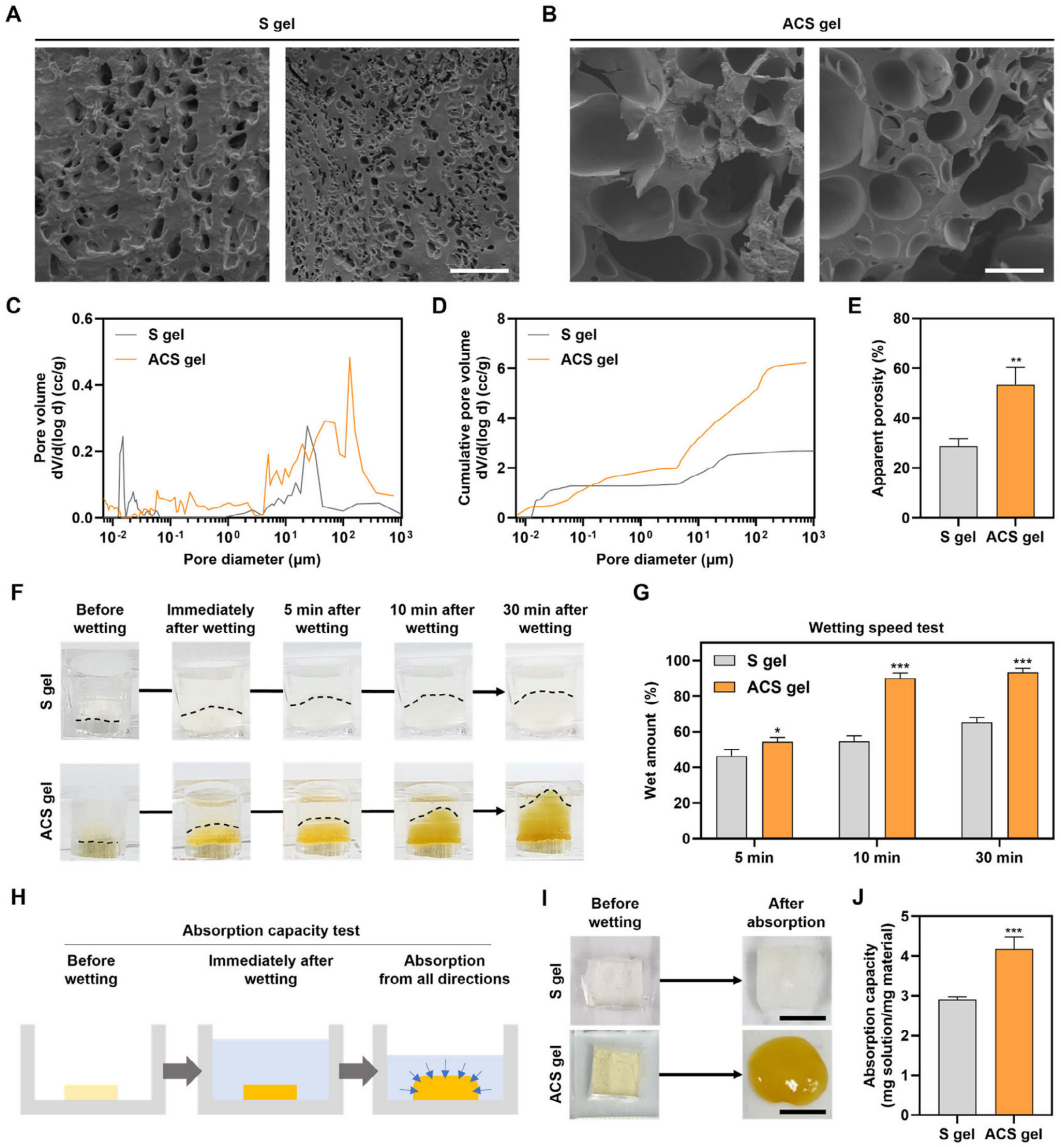
Improved absorbent properties of the ACS‐MP and the resultant ACS gel. SEM images of the internal structure of A) S gel and B) ACS gel (scale bars = 100 µm). C) Pore size distribution, D) cumulative pore volume, and E) porosity of the S gel and ACS gel as determined by mercury porosimetry (*n* = 3; ^**^ *p* < 0.01). F) Side views of the stacked S‐MPs (upper row) and ACS‐MPs (bottom row) before and after wetting the particles with BSA solution from the upper side. G) Quantification of the percentage of wet particles in the stacked particles at 5, 10, and 30 min after wetting (*n* = 4; ^*^ *p* < 0.05, ^***^ *p* < 0.001). H) Schematic illustration of absorption capacity test of the MP and the resultant gel in BSA solution. I) Photographs of each MP before wetting and the resultant gel after 30 min in BSA solution (scale bars = 1 cm). J) Quantification of the absorbed solution per unit mass of each MP (*n* = 4; ^***^ *p* < 0.001).

In examining the internal structure of hydrogels, the hydrogel produced through the self‐gelation of S‐MPs after wetting (S gel) exhibited a closed and stacked structure consisting of tiny pores (**Figure** **4**A). This characteristic is likely attributed to the tight intermolecular interactions between the starch polymers, leading to low flexibility of the polymer chains and difficulty in interacting with water molecules. In contrast, ACS displays reduced intermolecular interaction due to decrease in the glucose repeating units in starch by random aldehyde modification and increased steric hindrance after catechol functionalization. This results in an increased flexibility of the polymer chains, allowing water molecules to permeate more easily into the ACS polymers. Consequently, ACS can interact with a greater amount of water molecules than native starch. As a result, the hydrogel formed through the self‐gelation of ACS‐MPs after wetting (ACS gel) exhibited a more porous internal structure with larger pores compared to the S gel (Figure 4B). In the assessment of the pore distribution in both hydrogels, we observed that the ACS gel contained a greater number of pores with larger sizes compared to the S gel (Figure 4C,D). Quantitatively, the porosity of the ACS gel was ≈1.86 times higher than that of the S gel (Figure 4E). The differences in the internal structure and porosity of the hydrogels are likely to significantly impact their liquid absorption speed and maximum absorption capacity, which are critical factors in determining the hemostatic performance of the resultant hydrogels.

To assess the wetting speed of each MP and the resulting hydrogels, an experimental setting was designed to allow liquid to pass only through vertically stacked MPs within a mold (Figure S6, Supporting Information). Upon adding the bovine serum albumin (BSA) solution onto the stacked particles, the BSA solution above the S‐MPs permeated only their interface, whereas the solution above the ACS‐MPs was rapidly absorbed into the upper layer beyond the interface (Figure 4F). A much larger amount of the solution was penetrated and absorbed in the ACS gel group than in the S gel group. Consequently, the ACS gel became swollen vertically owing to more rapidly absorbing a larger amount of solution compared to the S gel. This phenomenon might be attributed to the larger porosity of the ACS gel resulting from the enhanced capability of ACS chains to interact with water and the unique gelation kinetics of ACS‐MP. Quantitatively, the amount of wet particles in the ACS gel group was notably larger than that in the S gel group at each time point for comparison (Figure 4G). The saturation in the absorption of the ACS gel group was rapidly achieved within ≈10 min, while the S gel group did not reach absorption saturation up to 30 min. Given this observation, we anticipated that the wetting speed of the ACS‐MPs would be much faster than that of the S‐MPs. By employing an experimental setting that allows the hydrogel to absorb the BSA solution from all sides, the absorption capacity of both S gel and ACS gel was also assessed (Figure 4H). After complete immersion in the BSA solution for sufficiently absorbing the solution from all directions, the degree of swelling and moisture content of the ACS gel were larger than those of the S gel (Figure 4I). The amount of absorbed solution per unit mass of MP in the ACS gel group was ≈1.4 times greater than that in the S gel group (Figure 4J). These enhancements in the absorption speed and capacity of ACS‐MPs would facilitate the hemostatic performance of the resultant ACS gel on the defective tissues covered with body fluids and blood.

### 2.4 Enhanced Tissue‐Adhesiveness of ACS Gel

**Figure 5:**
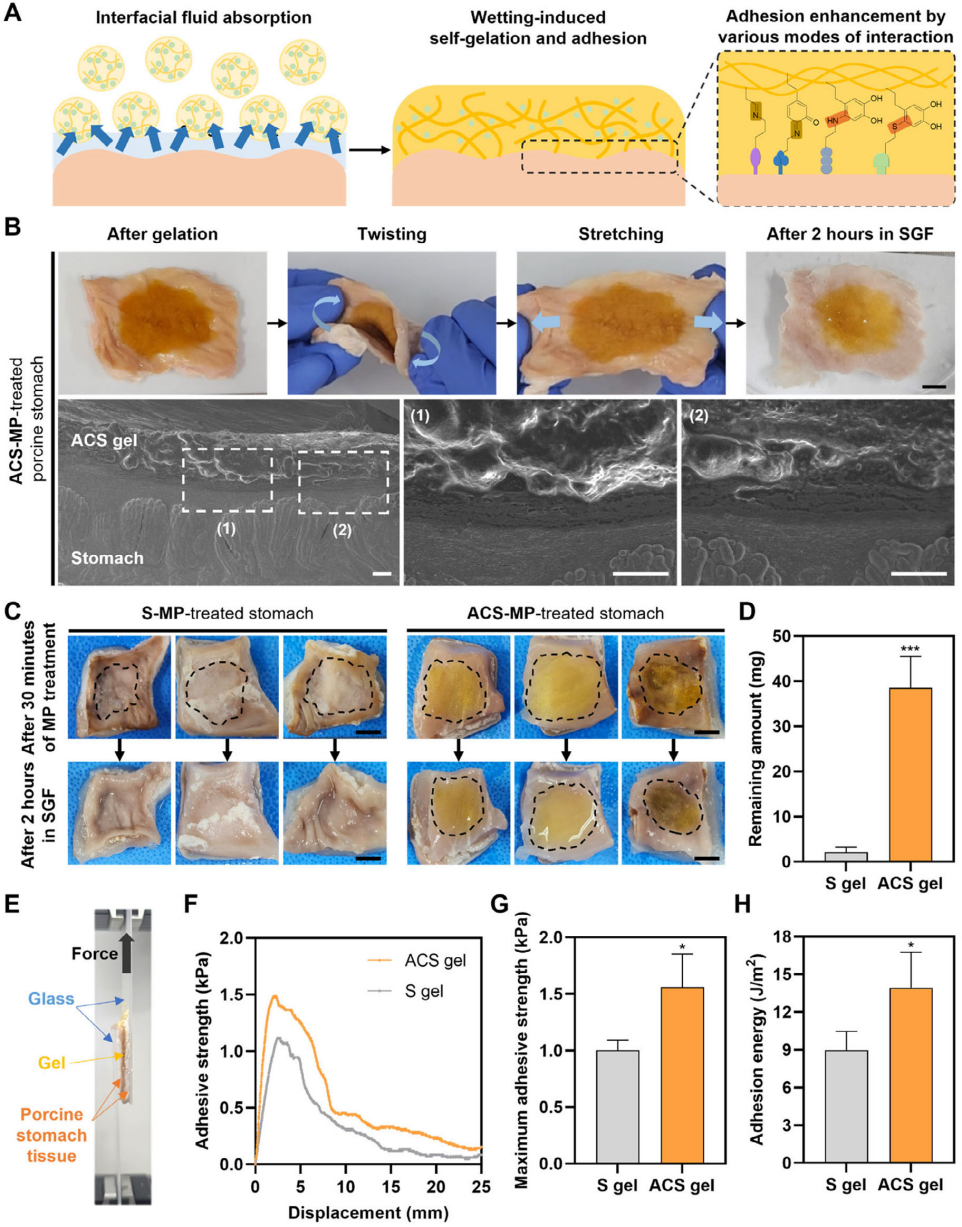
Enhanced tissue‐adhesiveness of the ACS‐MP and the resultant ACS gel. A) Schematic illustration of the physical and chemical mechanisms for robust wet‐tissue adhesion of the ACS‐MP. B) Photographs (scale bar = 1 cm) and SEM images (scale bars = 100 µm) of the ACS gel adhered onto the surface of porcine stomach tissue. C) Photographs of the stomach tissues treated with S‐MPs and ACS‐MPs after 30 min of MP treatment (upper row) and after 2 h of incubation in simulated gastric fluid (SGF) (bottom row). Black dotted lines indicate the boundary of the hydrogel on the tissue (scale bars = 1 cm). D) Quantification of the mass of the remaining hydrogel collected from the tissue surface after 2 h of incubation in SGF with shaking (*n* = 4; ^***^ *p* < 0.001). E) Photograph of the experimental setting for measuring tissue‐adhesiveness of the ACS gel between porcine stomach tissues through lap shear test using a UTM. F) Adhesive strength of the S gel and ACS gel to the surface of porcine stomach tissue measured through the lap shear test. G) Average maximum adhesive strength (peak value) and H) average adhesion energy (area under curve) of the S gel and ACS gel (*n* = 5; ^*^ *p* < 0.05).

The highly absorbent ACS‐MPs can act as a dry adhesive that absorbs and removes the fluid between tissue and MPs (**Figure** **5**A). ACS was designed to feature both catechol moiety, a critical functional group responsible for strong underwater adhesion seen in mussels, and aldehyde moiety with binding affinity to the amines abundantly present in biological tissues through Schiff base linkage.^[^ ^19^ ^]^ Thus, exposure of ACS‐MPs to the interfacial fluid induces their self‐gelation and adhesion to the tissue via various interactions between them (Figure 5A).^[^ ^20^ ^]^ Consequently, the resulting ACS hydrogel can stably adhere to tissues covered with biological fluids. Additionally, the micro‐scale size of ACS‐MPs allows for tight contact with the curved and irregular shapes of tissue surfaces.

To assess the tissue‐adhesiveness of the ACS gel, the ACS‐MP was applied to the wet surface of the stomach tissue, which is one of the highly stretchable tissues and also has curvy and irregular tissue surfaces. After gelation, the resulting hydrogel could stably adhere to the stomach tissue surfaces even while the tissue was being twisted and stretched (Figure 5B; Video S2, Supporting Information). Moreover, the ACS gel remained on the tissues after immersion in simulated gastric fluid (SGF) for 2 h. Scanning electron microscopy (SEM) observations revealed that the ACS gel tightly adhered to the curvy surfaces of the stomach tissue. To compare the tissue‐adhesiveness of ACS‐MP and S‐MP, the same amount of each particle was applied to the stomach tissue, and then the resulting hydrogels with the tissues were immersed in SGF with shaking (Figure 5C). After 2 h, almost all of the S gel disappeared, but the ACS gel was well maintained on the tissue surface. When measuring the remaining amount of each hydrogel, tiny amounts of S gel remained, whereas significant amounts of ACS gel were retained (Figure 5D). Although both MPs could form hydrogels and adhere to the tissue surface, these results may be attributed to differences in their gelation pattern and kinetics. While all ACS‐MPs could be integrated together and form a uniform stable hydrogel, only part of the S‐MPs could participate in the gelation process, making the formed S gel unstable and prone to disintegration under physical stress, such as shaking in SGF. Besides, the S gel adhered to the tissue surface mainly via the abundant hydroxyl groups in the starch, while the catechol and aldehyde groups in the ACS can additionally contribute to the tissue‐adhesiveness of the formed ACS gel.

To further confirm the enhanced adhesiveness of the ACS gel, we compared the adhesive strengths of both hydrogels on porcine stomach tissues using a lap shear test (Figure 5E,F). The maximum adhesive strength of the ACS gel was ≈1.55 times higher than that of the S gel (Figure 5G). This suggests that the forces contributing to interfacial adhesion in the ACS gel were strengthened by additional interactions involving catechol and aldehyde groups. Likewise, the adhesion energy of the ACS gel was higher than that of the S gel (Figure 5H). These results demonstrate that chemical modifications in the ACS not only enhance the interfacial interaction between the gel and tissues but also improve cohesiveness and efficiency in energy dissipation, thereby significantly increasing the tissue‐adhesiveness of the ACS gel.

### 2.5 Excellent Biocompatibility of ACS Gel

**Figure 6:**
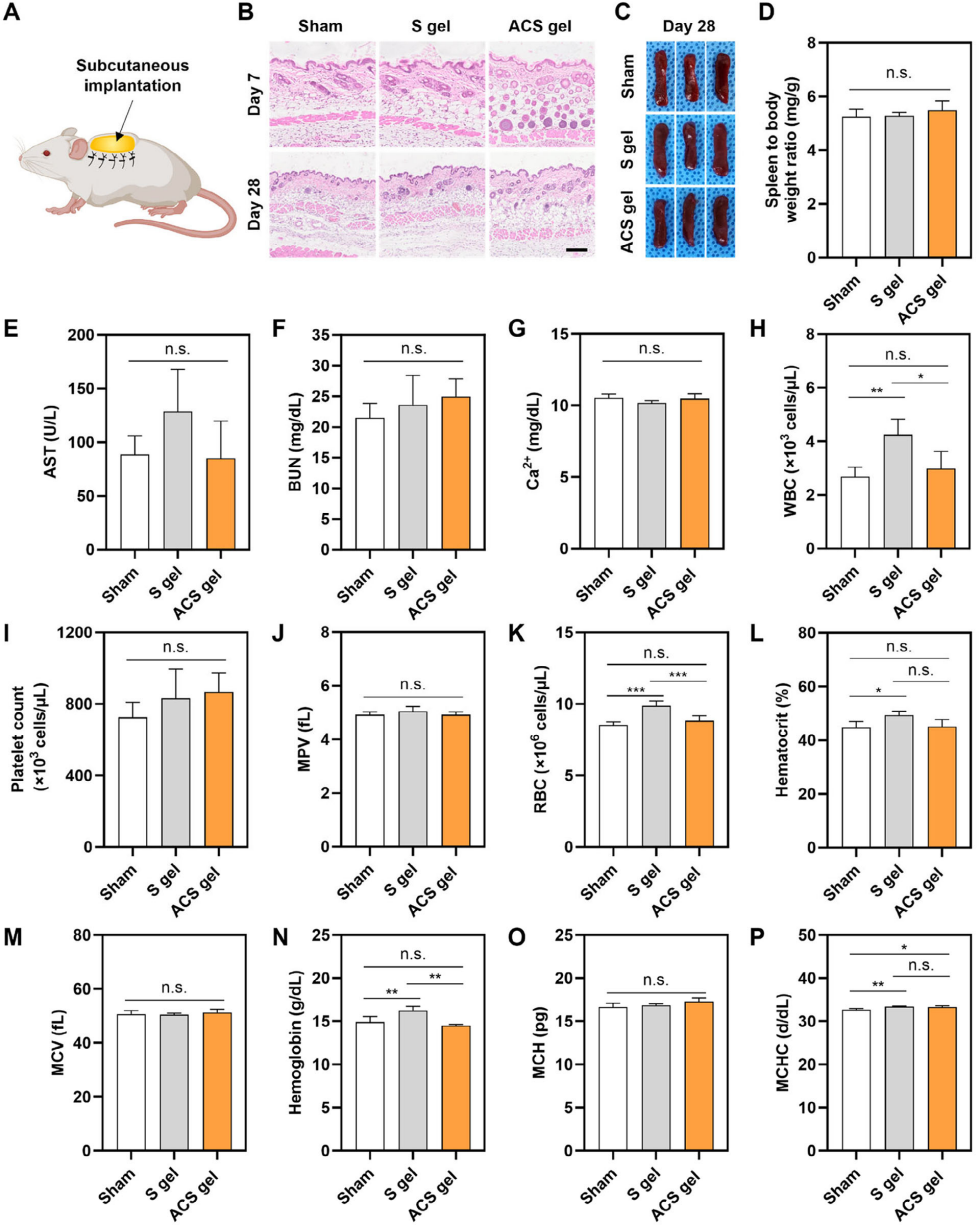
In vivo biocompatibility of the ACS gel in mouse subcutaneous tissue. A) Schematic illustration of the in vivo biocompatibility evaluation of the subcutaneously implanted ACS gel. B) H&E‐stained images of the subcutaneous tissues 7 and 28 days after implantation of the starch‐based gels (S gel group and ACS gel group) and without gel implantation (Sham group) (scale bar = 100 µm). C) Photographs of the spleen harvested from the mice in the Sham, S gel, and ACS gel groups. D) The weight ratio of the spleen to the body (mg/g) in the Sham, S gel, and ACS gel groups 28 days after the gel implantation (*n* = 3). Blood chemistry analysis for quantification of E) aspartate transferase (AST), F) blood urea nitrogen (BUN), and G) calcium ion (Ca^2+^) in the blood samples collected from the mice in the Sham, S gel, and ACS gel groups 28 days after gel implantation (*n* = 4; n.s. indicates not statistically significant). Hematological analysis for quantification of H) white blood cell (WBC), I,J) platelet‐related parameters (MPV: mean platelet volume), K–M) red blood cell‐related parameters (RBC: red blood cell, MCV: mean corpuscular volume), and N–P) hemoglobin‐related parameters (MCH: mean corpuscular hemoglobin, MCHC: mean corpuscular hemoglobin concentration) in the blood samples collected from the mice in the Sham, S gel, and ACS gel groups 28 days after gel implantation (*n* = 4; ^*^ *p* < 0.05, ^**^ *p* < 0.01, and ^***^ *p* < 0.001).

To investigate the biocompatibility of starch‐based hydrogels, both S gel and ACS gel prepared by treating each MP with phosphate‐buffered saline (PBS) were implanted into the subcutaneous tissue of mice (**Figure** **6**A). After 7 and 28 days of implantation, the subcutaneous tissues and major organs (heart, spleen, liver, kidney, and lung) were harvested and analyzed. In histological observations using hematoxylin and eosin (H&E) staining, the skin tissues covering the subcutaneously implanted hydrogels in the S gel group and ACS gel group did not show any pathological abnormalities like inflammation, necrosis, or metaplasia, which was similar to the skin tissues in the sham group (with only surgery without gel implantation) (Figure 6B). This indicates that starch‐based hydrogels did not evoke local toxicity or adverse effects. Furthermore, the appearance of the spleens harvested from the mice in S gel and ACS gel groups at day 28 did not show differences from those from the sham group (Figure 6C). Indeed, there was no statistically significant difference in the weight ratio of the spleen to the body among all groups (Figure 6D). These results suggest that the implanted starch‐based hydrogels did not induce any problematic local or systemic immune responses.

In morphological comparisons of major organs among all groups, all the tissues harvested on days 7 and 28 did not exhibit apparent tissue damage or pathological changes (Figure S7, Supporting Information). Blood biochemistry analysis on days 7 and 28 revealed that there were no significant differences in the functions of the liver (aspartate transferase; AST), kidney (blood urea nitrogen; BUN), and heart (Ca^2+^) between the sham group and ACS gel group (Figure 6E–G; Figure S8A–C, Supporting Information). Notably, the Ca^2+^ included for crosslinking ACS gel did not affect the level of blood Ca^2+^ in the body. In the hematological analysis, the ACS gel group did not show significant differences from the sham group in the levels of white blood cells, platelets, red blood cells, and hemoglobin in the blood (Figure 6H–P; Figure S8D–L, Supporting Information). Overall, our data demonstrates the excellent biocompatibility of the ACS gel, indicating its great potential for clinical translation.

Given that starch‐based hydrogels can undergo biodegradation through digestion processes, the ACS gel would be a promising candidate for biomedical applications in the gastrointestinal tract (GIT). We confirmed that both S gel and ACS gel could be degraded in PBS supplemented with amylase (Figure S9, Supporting Information). The starch‐based materials are enzymatically broken down by α‐amylase into maltose or dextrin, both of which are commonly encountered in everyday life and are well known for their biocompatibility and safety in the human body.^[^ ^21^ ^]^ These degradation products can be further metabolized by other enzymes into glucose, ensuring the biosafety of the materials’ primary degradation pathway under in vivo conditions. Dopamine, another key component of the ACS, is not susceptible to amylase‐mediated degradation. As dopamine is an endogenous compound naturally present in the human body, it has a significantly lower risk of triggering immune reactions or toxicity compared to artificial chemical crosslinkers.^[^ ^22^ ^]^ Dopamine can be metabolized through various physiological pathways or excreted via the renal system, further supporting its in vivo biosafety.^[^ ^23^ ^]^ Indeed, the biosafety of dopamine‐functionalized biomaterials has been extensively studied and validated in a lot of previous research.^[^ ^24^ ^]^ Furthermore, since dopamine is covalently conjugated to starch in ACS‐MPs, it is less likely to exist in a free‐form state after degradation of ACS gel. The covalent attachment of dopamine in our material may minimize excessive release of the free‐form of dopamine, thereby mitigating its potential safety concerns.

**Figure 7:**
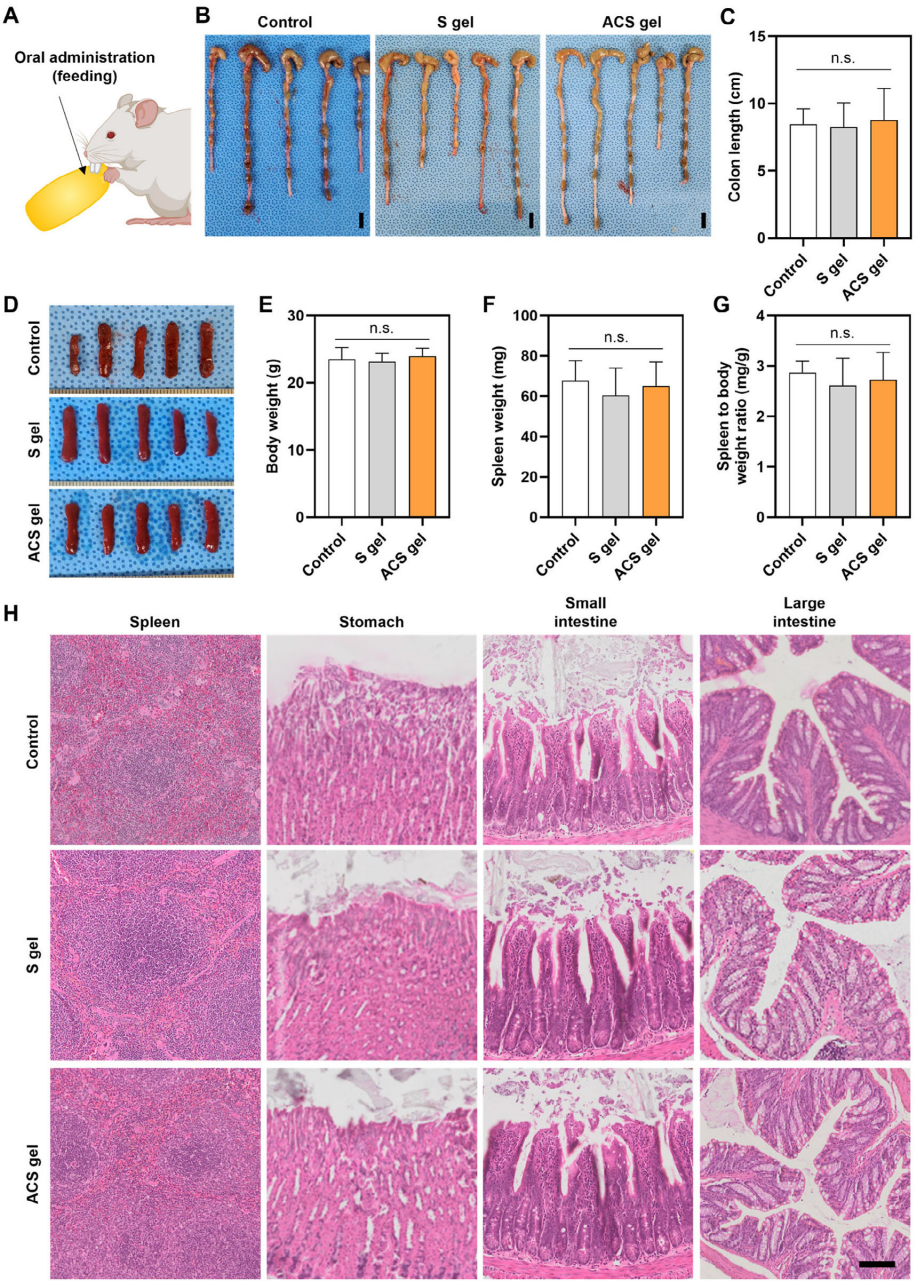
In vivo biocompatibility of the orally administrated ACS gel to mouse. A) Schematic illustration of the in vivo biocompatibility evaluation of the orally administrated ACS gel to mouse. B) Photographs and C) length of the colon harvested from the mice with oral administration of starch‐based gels (S gel group and ACS gel group) and only normal mouse diet without gel administration (control group) after 7 days of administration (*n* = 5, n.s. indicates not statistically significant. Scale bars = 1 cm). D) Photographs of the spleen harvested from the mice in the control, S gel, and ACS gel groups on day 7. E) Body weight, F) spleen weight, and G) weight ratio of spleen to body (mg/g) in the control, S gel, and ACS gel groups at day 7 (*n* = 5). H) H&E‐stained images of the spleen and major organs in the GI tract (stomach, small intestine, and large intestine) harvested from the mice in the control, S gel, and ACS gel groups at day 7 (scale bar = 100 µm).

To further investigate the biocompatibility of starch‐based gels in the GIT, S gel and ACS gel were orally administered to the mice as a part of the diet (**Figure** **7**A). After seven days of feeding, we compared the appearance of the colons harvested from the mice in all groups (Figure 7B). The mice with hydrogel feeding (S gel and ACS gel groups) did not show differences from the control group (fed with only normal mouse diet) in the shape and length of the colons. There was no statistically significant difference in the average length of the colons between starch‐based gel groups and the control group (Figure 7C). Furthermore, the appearances of the spleens harvested from the mice of all groups were quite similar (Figure 7D), and there were no significant changes in their body weight, spleen weight, and the ratio of spleen to body weight (Figure 7E–G). In H&E staining of the spleen and GIT (stomach, small intestine, and large intestine), the organs of the S gel and ACS gel groups did not exhibit any pathologically abnormal states (Figure 7H). These results suggest that the starch‐based hydrogels, especially the ACS gel, demonstrated no immunogenicity and toxicity when applied to GIT, indicating its promising potential as a highly biocompatible bio‐adhesive hydrogel for biomedical applications to various tissues, including the GIT, such as hemostatic agents and tissue sealants.

Additionally, we tested the feasibility of the starch‐based hydrogels as a drug delivery deposit. Drugs can be loaded into the starch‐based hydrogels by simply adding the drugs to the mixture of starch and calcium salt during fabrication of starch‐based MPs. As an example of anti‐inflammatory drug, dexamethasone encapsulated in the starch hydrogels was sustainedly released under enzymatic degradation conditions with amylase (Figure S10, Supporting Information), demonstrating their potential as a drug delivery system for GIT. Notably, the ACS gel showed prolonged drug release profiles compared to the S gel, which can be attributed to the presence of the catechol groups that can interact with various functional groups in drugs, allowing more effective drug encapsulation and delivery in the ACS gel.^[^ ^25^ ^]^

### 2.6 Effective Hemostasis of ACS‐MP in Acute Mass Bleeding

The micrometer size of the ACS‐MP (≈7.6 µm) offers significant advantages for application in irregularly shaped and deep tissue defects. Furthermore, owing to the enhanced tissue‐adhesiveness by the presence of catechol and aldehyde groups, the ACS gel would not only physically fill and block a wounded site but also bind to various blood components, including blood cells, platelets, and blood clotting factors and mediators, thereby facilitating robust hemostasis.^[^ ^26^ ^]^ The calcium ions included in the ACS gel can also play a crucial role in the blood coagulation cascade.^[^ ^27^ ^]^ More importantly, the improved absorption capability of the ACS gel would significantly enhance the accumulation of blood components, further contributing to a highly effective clotting process.^[^ ^28^ ^]^ Therefore, the ACS gel is expected to exhibit outstanding hemostatic performance.

**Figure 8:**
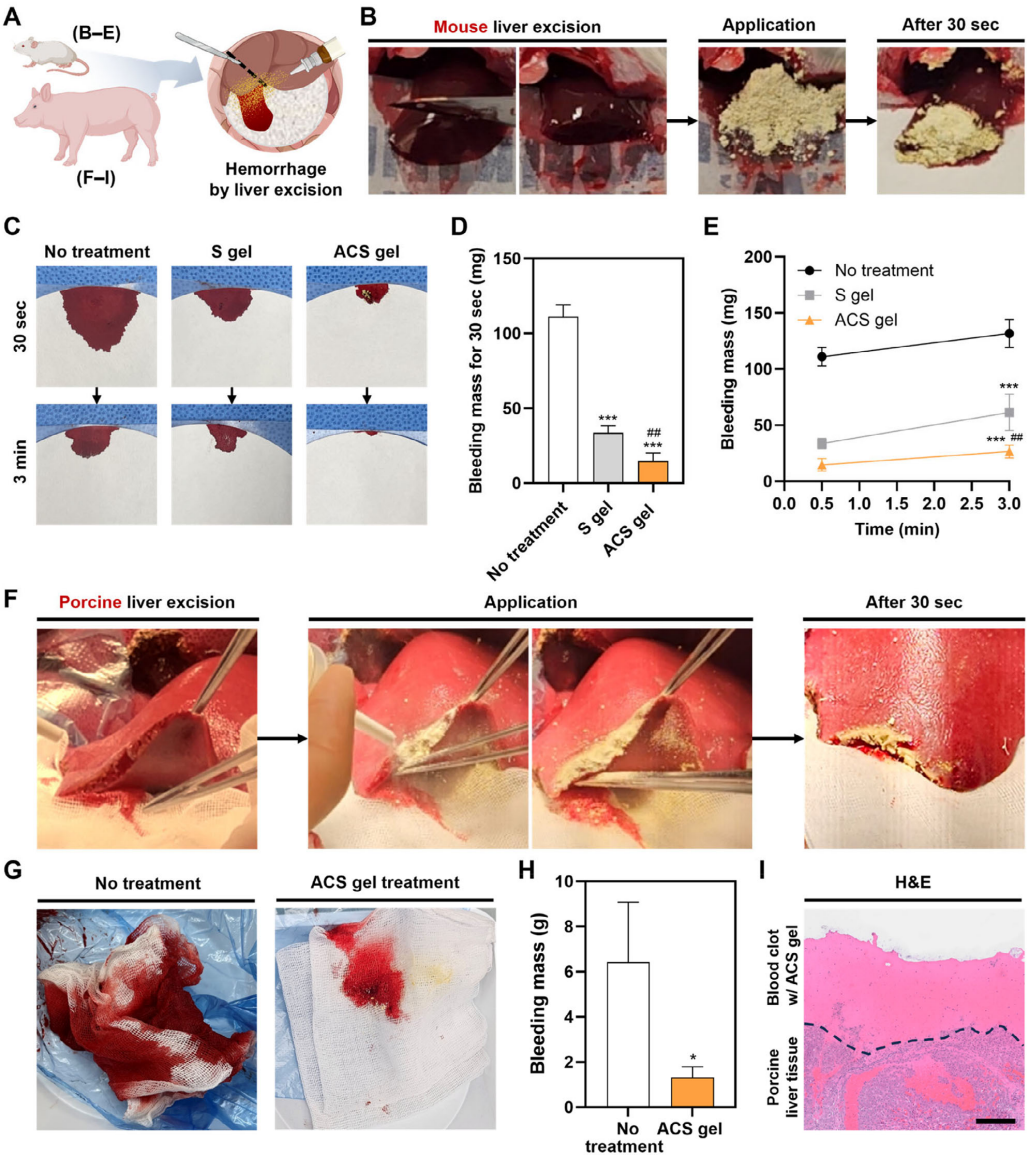
In vivo hemostatic capability of ACS‐MP in mouse and porcine models. A) Schematic illustration of hemorrhage models by liver excision in mice B–E) and pigs F–I). B) Photographs of a mouse liver excision‐induced hemorrhage model and the treated ACS‐MPs onto the cross‐section of bleeding tissue immediately and 30 s after application. C) Photographs of the filter papers that absorbed blood released from the wounds without any treatment (no treatment group) and with the treatment of S‐MPs (S gel group) and ACS‐MPs (ACS gel group) at 30 s and 3 min. D) Quantification of the bleeding mass measured for 30 s after MP treatments (*n* = 4; ^***^ *p* < 0.001 vs no treatment group, ^##^ *p*< 0.01 vs S gel group). E) Cumulative bleeding mass in no treatment, S gel, and ACS gel groups at 30 s and 3 min (*n* = 4; ^***^ *p* < 0.001 vs no treatment group, ^##^ *p* < 0.01 vs S gel group). F) Photographs of a porcine liver excision‐induced hemorrhage model and the treated ACS‐MPs onto the cross‐section of bleeding tissue during application and 30 s after application. G) Photographs of the gauzes that absorbed blood released from the wounds in the no treatment and ACS gel groups at 5 min after bleeding induction. H) Quantification of the bleeding mass measured at 5 min after ACS‐MP treatment (*n* = 3; ^*^ *p* < 0.05). I) H&E staining image of the porcine liver tissue after hemostasis in the ACS gel group. The black dotted line indicates a boundary between the liver tissue and the blood clot with the ACS gel (scale bar = 200 µm).

We assessed the hemostatic performance of the ACS gel in small and large animal models–mouse and porcine liver hemorrhage models induced by excision (**Figure** **8**A). In the mouse liver excision model, bleeding was induced by cutting the liver using a surgical blade. Treatment with the ACS‐MP for hemostasis appeared to stop the bleeding within 30 s (Figure 8B). Observing the filter papers to absorb the released blood and measure the bleeding amount, we found that the papers in the no treatment (NT) group and the S gel group at 30 s absorbed a larger amount of blood compared to the ACS group (Figure 8C). The bleeding mass in both S‐MP and ACS‐MP‐treated groups was significantly reduced compared to the NT group, indicating the effectiveness of both MPs for hemostasis in acute mass bleeding (Figure 8D). However, the bleeding in the S gel group appeared to continue over 3 min, while the bleeding almost stopped in the ACS gel group (Figure 8C,E). These results are attributed to differences between the S‐MP and ACS‐MP regarding gelation pattern, kinetics, absorption capacity, and adhesive functional groups. Since the applied S‐MPs gelled immediately upon contact with the blood and could not absorb the blood quickly, the formed S gel could not sufficiently adhere to and block the entire region of the bleeding site, leading to inadequate hemostasis. In contrast, the ACS‐MPs applied to the bleeding region gelled rapidly (within 3–4 s) while progressively swollen with high blood absorption, achieving complete hemostasis with tight adhesion on the injured bleeding tissue.

To further assess the hemostatic capability of the ACS gel in a large animal for preclinical study, we employed the porcine liver hemorrhage model. Following heparinization and excision of the liver to induce substantial bleeding, ACS‐MPs were applied to the bleeding site (Figure 8F; Video S3, Supporting Information). Remarkably, after only 30 s of application, the bleeding appeared to be completely halted, and the applied ACS‐MPs adhered well to the bleeding region with excision. When we checked the gauze to absorb the released blood in each group, the ACS group was slightly wet from only bleeding immediately after excision, whereas the NT group was soaked with the blood from the unstopped bleeding (Figure 8G). The bleeding was effectively suppressed by the treatment with the ACS gel (Figure 8H). H&E‐stained image of the defective tissue treated with the ACS gel showed the ACS gel aggregated with the blood to facilitate robust clotting and tightly adhered to the incised tissue (Figure 8I), thereby completely blocking the bleeding site and inducing effective hemostasis. These results suggest that the ACS gel holds great potential as a hemostatic agent capable of adequately controlling bleeding in humans.

Our ACS‐MPs have several comparative advantages as a hemostatic agent over existing commercial products from multiple perspectives. One of the most widely used commercial hemostatic agents in clinical practice is fibrin glue, like TISSEEL, which mainly consists of fibrinogen and thrombin proteins. Due to its protein‐based composition, fibrin glue has relatively high production costs and requires strict storage and preservation conditions. Furthermore, since it operates via the biological interaction between fibrinogen and thrombin, its application usually necessitates a dual‐barrel syringe to facilitate mixing and delivery. In contrast, the ACS‐MPs are primarily composed of starch, making it highly cost‐effective and less sensitive to temperature variations compared to protein‐based hemostatic agents. This facilitates efficient storage and transportation. Additionally, unlike fibrin glue, the ACS‐MPs function autonomously without the needs for mixing process, additional crosslinking agents, or specialized dual‐barrel syringes. Simple application of the ACS‐MPs directly to the bleeding site triggers self‐activation, allowing it to be effectively used across a broad range of surgical settings. These features of the ACS‐MPs offer significant comparative advantages over fibrin‐based hemostatic agents in terms of cost and ease of use.

Beyond cost‐effectiveness, convenience, and usability, the ACS‐MPs may also exhibit superior hemostatic performance in massive bleeding scenarios, particularly when compared to fibrin glue products. Through a comprehensive literature investigation, we found that commercial fibrin glue products may not be sufficiently effective in massive bleeding scenarios, particularly in liver hemorrhage models. For instance, in a puncture‐induced mouse liver hemorrhage model, TISSEEL was tested for hemostatic performance. A continuous increase in bleeding mass was observed until 2.5 min, and the cumulative bleeding mass reached ≈80 mg at the final timepoint, suggesting ineffective bleeding control.^[^ ^3b^ ^]^ In contrast, in an excision‐induced mouse liver hemorrhage model that shows a similar level of massive bleeding, our ACS‐MPs induced highly effective hemostasis, with a final bleeding mass of ≈26.5 mg at 3 min (Figure 8), which is significantly lower than that observed with TISSEEL. Similarly, in a large‐animal incision‐induced porcine liver hemorrhage model, Fibingluraas did not achieve hemostasis by the final measurement time point (180 s).^[^ ^29^ ^]^ At that point, the average bleeding mass remained ≈2.584 g, suggesting inadequate bleeding control. In our study using an excision‐induced porcine liver hemorrhage model, the ACS‐MPs achieved complete hemostasis within 30 s, and the final bleeding mass at 5 min was ≈1.31 g (Figure 8), which is substantially lower than that observed with Fibingluraas. Although we did not conduct direct comparison studies under identical experimental conditions and hemorrhage models, these analyses based on previous literature may suggest that the ACS‐MPs exhibit superior hemostatic performance in massive bleeding scenarios to fibrin glue products. In our hypothesis, the fibrin glue applied in a liquid form may be diluted by the large volume of blood or may fail to effectively seal the bleeding site because it does not function by absorbing blood. In contrast, the ACS‐MPs possess several unique advantages to effectively address these challenges in massive bleeding scenarios. Their ability to rapidly absorb blood and form an adhesive hydrogel barrier enables them to effectively achieve hemostasis in such conditions.

Apart from fibrin glue, several commercial hemostatic powders are also available in clinical situations. One of the most well‐known and widely used powder‐type hemostatic agents in clinical practice is Arista, which shares certain advantages with our ACS‐MPs, including cost‐effectiveness and ease of use, particularly as both are starch‐based. Arista consists of microporous particles made from chemically crosslinked starch, but it does not actively induce interactions between particles or adhesion to tissue surfaces. Instead, Arista functions by absorbing and concentrating blood components, thereby enhancing natural hemostasis. In comparison, our ACS‐MPs offer a distinct mechanism of action in hemostasis owing to chemical modifications such as partial oxidation and catechol functionalization. The ACS‐MPs also rapidly absorb the blood upon contact with the bleeding site, allowing efficient access to the surface of defective tissue. Simultaneously, they undergo ultrafast self‐gelation through interparticle interactions, forming a stable hydrogel directly on the bleeding site. Furthermore, leveraging the excellent tissue‐adhesive properties of the ACS‐MPs owing to the presence of catechol groups, the resultant hydrogel can firmly adhere to the tissue surface, serving as a protective barrier to tightly seal the bleeding site. Similar to Arista, the ACS‐MPs exhibit highly absorbent properties, which facilitates the concentration of blood components and natural hemostasis process. Given these multiple modes of action, including highly absorbent properties, ultrafast self‐gelation, and tissue‐adhesiveness, it is anticipated that the ACS‐MPs demonstrate better hemostatic performance than existing commercial starch‐based hemostatic powders.

Based on comparative analysis with previous literatures, we assessed the general performance levels of our ACS‐MPs and existing commercial powder‐type hemostatic agents. In a punch‐induced porcine liver hemorrhage model, the hemostatic performance metrics of three powder‐type hemostatic agents (Arista, PerClot, and Surgicel Powder) were analyzed in terms of hemostasis success rate within 10 min and time‐to‐hemostasis (TTH).^[^ ^30^ ^]^ Both Arista and Surgicel Powder achieved a 100% success rate, whereas PerClot showed occasional failure cases. In terms of TTH, except for Surgicel Powder (≈30 s), the other agents required a considerably longer time, ranging from ≈285 to 438 s. Another study conducted under similar hemorrhage conditions reported TTH values of 1.71, 3.26, 3.27, and 4.46 min for Surgicel Powder, Arista, PerClot, and 4DryField, respectively.^[^ ^31^ ^]^ Additionally, in an excision‐induced porcine liver hemorrhage model treated with Arista, hemostasis was not achieved within 10 min.^[^ ^32^ ^]^ In contrast, under a similar excision‐induced porcine liver hemorrhage model, our ACS‐MPs achieved hemostasis within ≈30 s (Figure 8). Thus, we can infer that in massive bleeding scenarios, the ACS‐MPs may exhibit a hemostatic performance comparable to Surgicel Powder and superior to the three starch‐based powder products (Arista, PerClot, and 4DryField). While these comparisons were not conducted under identical conditions, our approach can provide a valuable insight and future direction for the development of next‐generation powder‐type hemostatic agents in clinical settings.

## 3 Conclusion

In this study, we developed a bio‐inspired starch‐based MP as a hemostatic biomaterial by incorporating aldehyde and catechol groups into starch polymers and subsequently freeze‐milling the calcium‐intercalating starch gels. Although the native starch‐based MP and resultant hydrogel (S gel) exhibited rapid self‐gelling and good absorption capabilities because of the inherent physical and chemical properties of starch, they underperformed in tissue‐adhesiveness under wet conditions and in hemostatic performance due to their limited interaction with water molecules. Partial oxidation of the native starch not only reduced intermolecular interactions in starch and loosened its highly packed structures, allowing water molecules to easily permeate into the polymers, but also introduced aldehyde groups into the starch polymers, contributing to the reinforcement of interactions with biomolecules. Additional modification of partially‐oxidized starch polymer with the catechol group contributed to the steric hindrance among the polymer chains, improving water absorption capabilities and further strengthening tissue‐adhesiveness of the starch hydrogel. As a result, the ACS‐MPs can more rapidly absorb aqueous solutions than unmodified S‐MPs, leading to ultrafast and uniform self‐gelling to form a highly stable hydrogel with integrity. The resultant ACS hydrogel with highly absorbent properties exhibited superior tissue‐adhesiveness onto the irregularly shaped, wet tissue surfaces compared to the native starch hydrogel. We also confirmed that the ACS hydrogels have excellent biocompatibility without local and systemic toxicity and immune responses. Owing to the catechol group that can bind to various functional groups in drugs, the ACS hydrogel would be considered a promising sustained drug delivery system.

Finally, we validated that the highly absorbent and adhesive ACS‐MPs enable the in situ formation of clot‐like constructs on the bleeding site via rapid absorption of blood and robust accumulation of blood components and coagulation factors. These processes not only physically block the wound but also promote coagulation, achieving effective hemostasis in a liver dissection‐induced mass bleeding model in mice and pigs. Given several advantages of ACS‐MP, including the ultrafast and efficient self‐gelling properties, highly absorbent properties, excellent biocompatibility, non‐compressible hemostatic capabilities, easy usage, and low cost, we believe that ACS‐MP would be a promising hemostatic material for effectively controlling acute mass bleeding in various tissues in clinical settings. Although we suggest that the ACS‐MPs present comparative advantages over existing commercial hemostatic agents via indirect comparison analyses, we acknowledge the necessity of direct comparative studies to comprehensively evaluate the performances and advantages of ACS‐MPs in future work. Owing to the outstanding tissue‐adhesiveness and biodegradability of ACS hydrogel, its application can be expanded to tissue sealing, wound healing, and drug delivery.

## 4 Experimental Section

### Synthesis of ACS Polymer

Aldehyde modification of starch was conducted according to the previously reported procedure with slight modifications.^[^ ^33^ ^]^ Starch from corn (unmodified waxy corn starch of essentially pure amylopectin containing only trace amounts of amylose; Sigma–Aldrich, St. Louis, MO, USA) was dispersed in triple distilled water (TDW) at a concentration of 100 mg mL^−1^ via vigorous agitation using a magnetic stirrer. An aqueous solution of sodium periodate (NaIO_4_; Sigma–Aldrich) was added dropwise to the starch solution at a 1:0.25 molar ratio of starch to NaIO_4_, and the mixture was vigorously stirred for 2 h at room temperature in the dark. Then, the unreacted NaIO_4_ was quenched by adding ethylene glycol to the reaction mixture and further stirring for 1 h in the dark. The unreacted chemicals and byproducts were removed through exhaustive dialysis using a dialysis membrane with an MW cut‐off of 6–8 kDa (Spectra/Por 1 RC Dialysis Membrane Tubing, Thermo Fisher Scientific, Waltham, MA, USA) against TDW for three days. The dialyzed solution was lyophilized and stored at 4 °C before use. The percentage of aldehyde‐modified glucose repeating units in the aldehyde‐modified starch was determined by measuring the content of the aldehyde group through an acid‐base titration method using NaOH, HCl, and phenolphthalein indicator (Sigma–Aldrich) as previously described,^[^ ^34^ ^]^ which was ≈40%.

For catechol conjugation, the aldehyde‐modified starch was dispersed in TDW at a concentration of 100 mg mL^−1^ via vigorous agitation. Then, dopamine hydrochloride (Sigma–Aldrich) was added to the solution at a 1:0.25 molar ratio of aldehyde‐modified starch to dopamine hydrochloride, and the mixture was vigorously stirred for 2 h to spontaneously induce Schiff base formation. The mixture was exhaustively dialyzed against TDW using the Spectra/Por 1 RC Dialysis Membrane to completely remove the unconjugated dopamine. After dialysis, the mixture was centrifugated at 1000 rpm for 5 min at room temperature, and the supernatant was removed. The remaining ACS was lyophilized and stored at 4 °C before use. The degree of substitution of dopamine per glucose repeating unit in the aldehyde‐modified starch was ≈11%, as determined by measuring the atomic percentage of carbon and nitrogen in the polymer through XPS analysis using a K‐alpha X‐ray photoelectron spectrometer (Thermo Fisher Scientific).

### Preparation of Starch‐Based MPs

The starch was evenly dispersed in TDW at a concentration of 200 mg mL^−1^ via vigorous agitation, and CaCl_2_ (Sigma–Aldrich) was added to the solution at a 2:1.567 mass ratio (≈1:1 molar ratio) of starch to CaCl_2_. The mixture was heated at 60 °C for 30 min with vigorous agitation and further heated at 85 °C for 15 min. When the mixture became slightly viscous after additional heating at 100 °C for 15 min, the mixture was poured into a petri dish. Then, the mixture was completely dried at 37 °C under a vacuum to produce dry S gel. The dry ACS gel was made via the same procedure using ACS instead of starch. The produced dry S gel or dry ACS gel was sliced into pieces a few millimeters in size. The pieces of dry gels were processed through cryogenic grinding using a cryogenic mill (6775 Freezer/Mill, SPEX SamplePrep, Metuchen, NJ, USA) with pre‐cooling for 2 min and grinding at 15 cps for 10 min for preparing starch‐based MPs. The fabricated starch‐based MPs were further lyophilized and stored at 4 °C before use.

### Characterization of Starch‐Based MPs

The morphology of starch‐based MPs was observed using a scanning electron microscope (SEM; JEOL‐7800F, JEOL Ltd., Tokyo, Japan) after pre‐treatment of platinum (coating regime: 20 mA current, 1 min) using a Cressington sputter coater 208HR (Cressington Scientific Instruments, Watford, UK). The size of the particles was determined by measuring the diameter of each MP in the acquired SEM images.

### In Vitro Biocompatibility Test of Starch‐Based Gels

In vitro biocompatibility was investigated in cell culture using an MP‐conditioned medium, according to the previously reported method, with slight modifications.^[^ ^35^ ^]^ The conditioned medium was prepared by incubating starch‐based MPs (S‐MP and ACS‐MP) at a concentration of 1 and 2 mg mL^−1^ in Dulbecco's modified Eagle medium (DMEM) supplemented with 10% fetal bovine serum (Thermo Fisher Scientific) and 1% penicillin/streptomycin (Thermo Fisher Scientific) at 37 °C for 24 h. DMEM medium without incubation of MPs served as a control. Human neonatal dermal fibroblasts (Thermo Fisher Scientific) were seeded onto cell culture plates at a density of 8000 cells cm^−2^. One day after the cell seeding, the fibroblasts were treated with the conditioned medium and further incubated at 37 °C for 24 h. Then, the cells were stained using a Live/Dead viability/cytotoxicity kit (Thermo Fisher Scientific) following the manufacturer's protocol and observed using a fluorescence microscope (IX73, Olympus, Tokyo, Japan). The cell viability was also evaluated quantitatively by measuring the mitochondrial activity of the cells in each conditioned medium‐treated group using a 3‐(4,5‐dimethylthiazol‐2‐yl)‐2,5‐diphenyltetrazolium bromide (MTT) assay (Sigma–Aldrich) and normalizing it to that of the control group (no treatment).

### Gelation and Characterization of Starch‐Based Gels

For preparation and characterization of starch‐based gels, self‐gelation of S gel and ACS‐MPs was induced with 4% (w/v) BSA solution as a substitute for body fluid. Unless otherwise noted, the starch‐based gel samples were prepared by applying 200 µl of BSA solution to 100 mg of MPs for uniform and sufficient wetting. The XPS spectrum of the gel was measured using a K‐alpha X‐ray photoelectron spectrometer (Thermo Fisher Scientific). The sample for the XPS analysis was prepared by lyophilizing the gel after complete gelation.

### Rheological Analysis

MCR 102 rheometer (Anton Paar, Ashland, VA, USA) was used for rheological analyses of starch‐based hydrogels. The storage modulus (G′) and loss modulus (G′′) of the starch‐based gels were measured using a frequency sweep test method at a frequency range of 0.1–10 Hz. The elastic modulus of the gel was determined by calculating the average of the measured storage modulus at 1 Hz. For investigation of gelation kinetics, the starch‐based MPs were evenly placed on the stage of the rheometer, and immediately after adding BSA solution onto the MPs, G′ and G′′ were measured using a time sweep test method for 60 s.

### Analysis of Porous Properties

After lyophilization of each starch‐based gel sample, their internal structures were observed using the JEOL‐7800F SEM after pre‐treatment of platinum (coating regime: 20 mA current, 1 min) using the Cressington sputter coater 208HR. The pore size distribution and porosity of the gels were investigated using a mercury intrusion porosimeter (Mercury Porosimeter PM33GT‐17, Quantachrome, Boynton Beach, FL, USA).

### Analysis of Absorption Properties

For the wetting speed test, a custom experimental setting that exposes only the upper side of the MPs stacked in the mold to the BSA solution, allowing the solution to pass through the MPs exclusively from top to bottom was employed. This setup is schematically illustrated in Figure S6 (Supporting Information). In detail, 300 mg of MPs were firmly stacked in the mold and temporally attached to the glass substrate, and then 1 mL of BSA solution was applied to the top of the stacked MPs. After 5, 10, and 30 min of absorption, the mold and the wet particles in a gel state were carefully removed together, and the weight of the remaining dry particles was measured. The wet amount (%) was calculated with (W_i_–W_d_)/W_i_ × 100, where W_d_ and W_i_ indicate the weight of the remaining dry MPs and the initial MPs, respectively.

For the absorption capacity test, 100 mg of MPs were firmly stacked in the mold and treated with 200 µl of BSA solution. After a brief wait for the gels to form stably, the mold was removed, and the gels were fully immersed in the BSA solution to absorb the solution from all directions. After 30 min of absorption, the weights of the gels were measured. The absorption capacity was calculated with (W_a_−W_i_)/W_i_, where W_a_ and W_i_ indicate the weight of the gel after absorption of the solution and the weight of the initial MPs, respectively.

### Tissue‐Adhesiveness Test

Porcine stomach tissues purchased from a local butcher shop were used to investigate the tissue‐adhesive properties of starch‐based gels. Each starch‐based MP was applied to the tissue surface, and 30 min waited to induce spontaneous gelation and adhesion. To investigate the interface between the gel and the stomach tissue, the cross‐section of the lyophilized gel‐tissue sample was observed using the SEM (JEOL Ltd.) after platinum coating. For investigation of the retention of the gel adhered onto the tissue surface, the gel with the tissue was immersed in SGF consisting of 0.2% (w/v) NaCl in 0.7% (v/v) HCl for 2 h with shaking. The remaining gel on the tissue surface was collected and lyophilized for measuring its weight to evaluate the retention of the gel. The adhesive strength of the gel to the tissue was investigated by a lap‐shear test using a universal testing machine (UTM, Mecmesin, MultiTest 2.5‐i, Slinfold, West Sussex, UK). The thinly sliced tissues were attached to the glass substrates using a commercially available adhesive (Loctite 401, Henkel, Düsseldorf, Germany), and the MPs were applied between the tissues. After 30 min to allow spontaneous gelation and adhesion of the applied MPs, the force was measured with the UTM by pulling the probe equipped on the UTM with a clip‐shaped customized jig for fixing the glass substrate at a speed of 50 mm min^−1^. Maximum adhesive strength and adhesion energy were determined by calculating the average value of the peak force and the area under the curve in the measured force‐distance curves, respectively.

### In Vivo Biocompatibility Test of Starch‐Based Gels

In vivo biocompatibility was investigated in mice, according to the experimental procedures approved by the Institutional Animal Care and Use Committee (IACUC) of Yonsei University (permit number: IACUC‐A‐202205‐1480‐01). The gels for subcutaneous implantation were prepared by treating 25 mg of the MPs with 50 µl of PBS. A 5‐week‐old mouse (ICR, female, Orient Bio, Seongnam‐si, Gyonggi‐do, Korea) was anesthetized by an intramuscular injection of a mixture of ketamine (100 mg kg^−1^, Yuhan, Seoul, Korea) and xylazine (20 mg kg^−1^, Bayer Korea, Ansan‐si, Gyonggi‐do, Korea). After shaving hair on the back of the mouse, a small incision was made on the back skin. The pre‐prepared S gel and ACS gel were implanted through the incision into the subcutaneous region, and then the incision was sutured and disinfected with betadine solution. Then, 200 µl of PBS was injected around the implantation site to prevent the drying of the surrounding tissues due to water absorption by the gel. The mouse that had only surgery and PBS injection without gel implantation was used as a sham group. After 7 and 28 days of implantation, the mice were euthanized, and their body and spleen weights were measured. Their skin, spleen, heart, liver, kidney, and lung tissues were also harvested, fixed with 10% formalin (Sigma–Aldrich), and embedded in paraffin for histological analysis. The embedded tissues were sectioned and stained with hematoxylin (Sigma–Aldrich) and eosin Y (Samchun Chemicals, Seoul, Korea). The stained samples were observed using a slide scanner (VS120‐S5‐W; Olympus). Blood samples collected from mice 7 and 28 days after gel implantation were used for blood biochemistry analysis and hematological analysis. The blood biochemical analysis for AST, BUN, and Ca^2+^ was conducted using DRI‐CHEM 4000i (Fuji Film, Tokyo, Japan). A complete blood count for hematological analysis was performed using HemaVet 950 (Drew Scientific, Miami Lakes, FL, USA).

For investigating the biocompatibility of the gels in the GIT, mice were fed daily with starch‐based gels and normal mouse diet mixed in an equal mass ratio. The mice that had only a normal diet were used as a control. It was carefully checked that the gels were consumed by the mice. After seven days of feeding, the mice were euthanized, and their body and spleen weights were measured. The lengths of the harvested colons from mice were also measured, and the spleen, stomach, small intestine, and large intestine were harvested, processed, and observed in the same way described above for histological analysis.

### In Vitro Degradation Test

For the in vitro degradation test, 100 mg of MPs were treated with 200 µl of PBS to form stable gels. Then, the gels were immersed in 1 mg ml^−1^ of α‐amylase (Junsei Chemical Co., Tokyo, Japan) solution at 37 °C to induce enzymatic degradation of the starch‐based gels. The degradation behaviors of the gels were investigated by visually observing the remaining gels 3 h after treatment with the enzyme solution.

### Drug Releasing Test

To encapsulate dexamethasone (Sigma–Aldrich) into MPs, dexamethasone was simply added to the starch mixture solutions containing starch or ACS and CaCl_2_ before pouring them into a mold during the procedure for preparing starch‐based MPs. The content of dexamethasone was determined to include 0.5 mg of dexamethasone per 150 mg of starch. To investigate the drug‐releasing capability of starch gels, 100 mg of MPs were treated with 200 µl PBS to form the drug‐loaded gels. Then, the gels were immersed in 0.5 mg mL^−1^ of α‐amylase solution or PBS at 37 °C, according to the previously reported study.^[^ ^36^ ^]^ The solutions were collected every day for 5 days while ensuring the collected solutions did not include the MPs. To quantify the amount of dexamethasone in the collected solution, an enzyme‐linked immunosorbent assay (ELISA) was conducted using a dexamethasone ELISA kit (Elabscience Bionovation Inc., Houston, TX, USA), according to the manufacturer's protocol.

### In Vivo Hemostatic Capability Test of Starch‐Based Gels

In vivo hemostatic capability was evaluated using a mouse and porcine liver hemorrhage model, according to the experimental procedures approved by the IACUC of Yonsei University (Approval number: IACUC‐A‐202205‐1480‐01) and Yonsei University Severance Hospital (approval number: 2023‐0230). For a small animal study, a 7‐week‐old mouse (ICR, female, Orient Bio) was anesthetized by an intramuscular injection of a mixture of ketamine (100 mg kg^−1^, Yuhan) and xylazine (20 mg kg^−1^, Bayer Korea). A small incision at the abdominal area in the mouse was made, and then the liver was taken out and placed on the filter paper. The liver was sliced ≈1 cm from the end of the liver lobe, exposing a cross‐section of ≈1.5 cm in width to induce mass bleeding. Subsequently, 50 mg of S‐MP and ACS‐MPs were applied to the bleeding cross‐section of the liver. The mice with no treatment served as a control (NT group). The amount of released blood was calculated by measuring the weight of filter papers that absorbed the blood at predetermined time points (30 s and 3 min). For a large animal study, the porcine liver hemorrhage model was employed. A pig (≈45 kg, Yorkshire, female, XP Bio, Anseong‐si, Gyeonggi‐do, Korea) was anesthetized by an intramuscular injection of the mixture consisting of alfaxalone (Alfaxan multidose, 1 mg kg^−1^, Jurox, Rutherford, NSW, Australia), medetomidine (Tomidine, 0.04 mg kg^−1^, Provet, Istanbul, Turkey), and azaperone (Stresnil, 2 mg kg^−1^, Elanco, Cuxhaven, Germany). Then, the anesthesia was maintained with 2−3 MAC of isoflurane (Ifran, Hana Pharm. Co. Ltd., Hwaseong‐si, Gyeonggi‐do, Korea), and the abdominal area was surgically opened. Before inducing bleeding, the pig was heparinized by intravenously injecting heparin (200 IU kg^−1^, JW Pharmaceutical, Gwacheon‐si, Gyeonggi‐do, Korea), and gauze for absorbing blood was placed under the liver. Then, the liver was sliced using operating scissors ≈1.5 cm from the end of the liver lobe, exposing a cross‐section of ≈5 cm in width to induce massive bleeding. The ACS‐MPs were applied to the bleeding liver using a commercially available spray applicator, covering the entire cross‐section. After 5 min of bleeding, the blood‐absorbed gauzes were weighed to calculate the bleeding mass, and the liver was harvested, processed, and observed as described above for histological analysis.

### Statistical Analysis

Quantitative data were expressed as mean ± standard deviation. Sample sizes were determined via power analysis (target power = 80%, α = 0.05, 0.01, and 0.001) using GraphPad Prism (GraphPad Software, CA, La Jolla, USA) to ensure sufficient statistical power. The sample size (*n*) for each statistical analysis was indicated in the figure legends. Statistical analysis was performed with the Students *t*‐test or one‐way analysis of variance (ANOVA) using GraphPad Prism. Results with *p*‐values < 0.05, 0.01, or 0.001 were considered statistically significant.

## Conflict of Interest

S.‐W. C., S. A., J. J., and D. J. J. are co‐inventors on a patent application (Korean Patent 10‐2023‐0104203, pending) related to aldehyde‐ and catechol‐modified starch (ACS) microparticles for hemostats used in the manuscript. S.‐W. C. is a chief technology officer (CTO) of CellArtgen Inc., Republic of Korea.

## Supporting information



Supporting Information

Supplemental Video 1

Supplemental Video 2

Supplemental Video 3

## Data Availability

The data that support the findings of this study are available from the corresponding author upon reasonable request.
