# Supplementary material for: Highly Absorbent Ultrafast Self‐Gelling Starch Microparticles for Robust Wet‐Tissue Adhesion and Instant Hemostasis
Source: Adv Sci (Weinh). 2025 Mar 24;12(19):2501857. doi: 10.1002/advs.202501857 (PMC12097133; doi:10.1002/advs.202501857)
Supplement: Supplementary file 1 — Supporting Information [file ADVS-12-2501857-s001.docx]

Supporting Information

Highly Absorbent Ultrafast Self-Gelling Starch Microparticles for Robust Wet-Tissue Adhesion and Instant Hemostasis

Soohwan An^†^, Jihoon Jeon^†^, Seung Yeop Han, Mi Jeong Lee, Tae-Gyeong Oh, Eun Je Jeon, Dong Jin Joo, and Seung-Woo Cho*

Dr. S. An, J. Jeon, S. Y. Han, M. J. Lee, T.-G. Oh, Prof. S.-W. Cho

Department of Biotechnology

Yonsei University

Seoul 03722, Republic of Korea

E-mail: [seungwoocho@yonsei.ac.kr](mailto:seungwoocho@yonsei.ac.kr)

S. Y. Han

Department of Biomaterials Science and Engineering

Yonsei University

Seoul 03722, Republic of Korea

Dr. E. J. Jeon, Prof. S.-W. Cho

CellArtgen Inc.

Seoul 03722, Republic of Korea

Prof. S.-W. Cho

Center for Nanomedicine

Institute for Basic Science (IBS)

Seoul 03722, Republic of Korea

Prof. D. J. Joo

Department of Surgery

Severance Hospital, Yonsei University College of Medicine

Seoul 03722, Republic of Korea

^†^These authors contributed equally to this work.

*Corresponding author: [seungwoocho@yonsei.ac.kr](mailto:seungwoocho@yonsei.ac.kr) (S.-W.C.)

Keywords: starch, ultrafast self-gelation, microparticle, absorbent & adhesive hydrogel, hemostasis


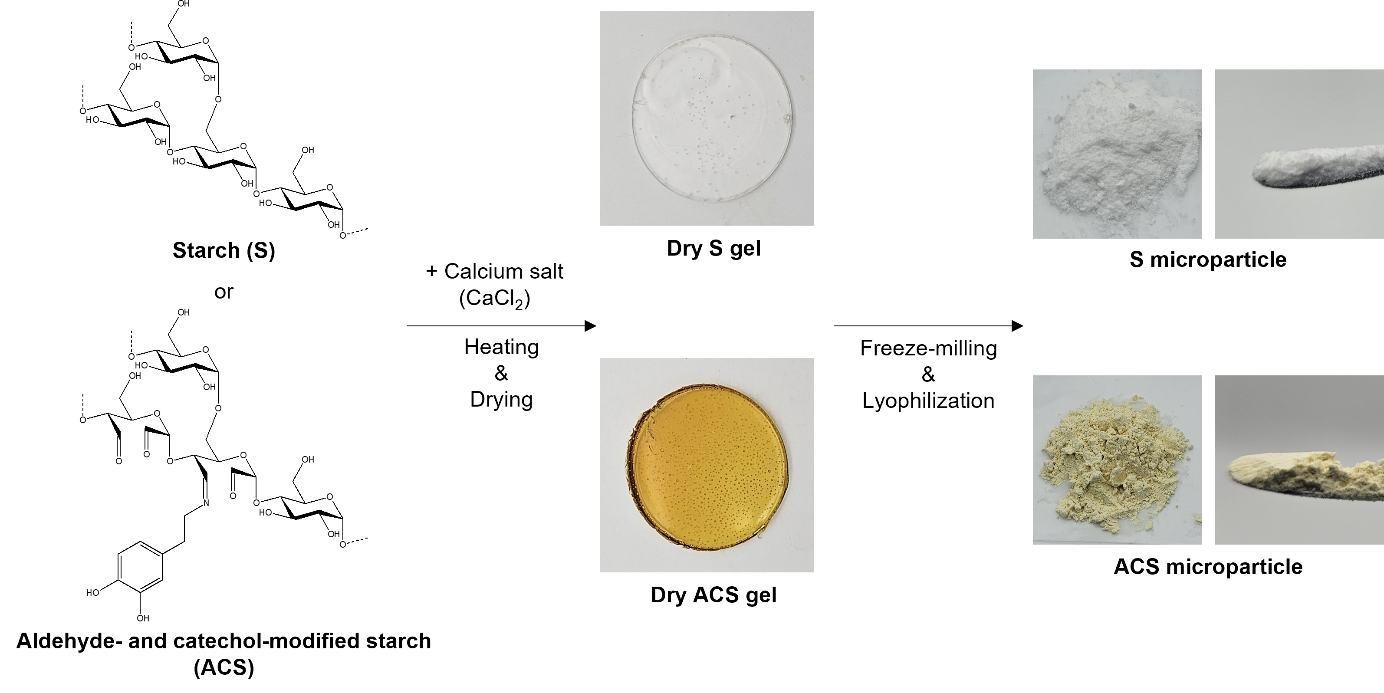


**Figure S1. Fabrication process of starch-based microparticles (MPs).** Starch (S) or aldehyde- and catechol-modified starch (ACS) was dispersed in water with vigorous mixing, and calcium salt was added to the solution. The mixture was heated, poured into a desired mold, and dried completely to form dry starch-based gels. The dry gels were freeze-milled and lyophilized to fabricate starch-based MPs. Detailed method and parameters for fabricating the MPs are described in the experimental section.


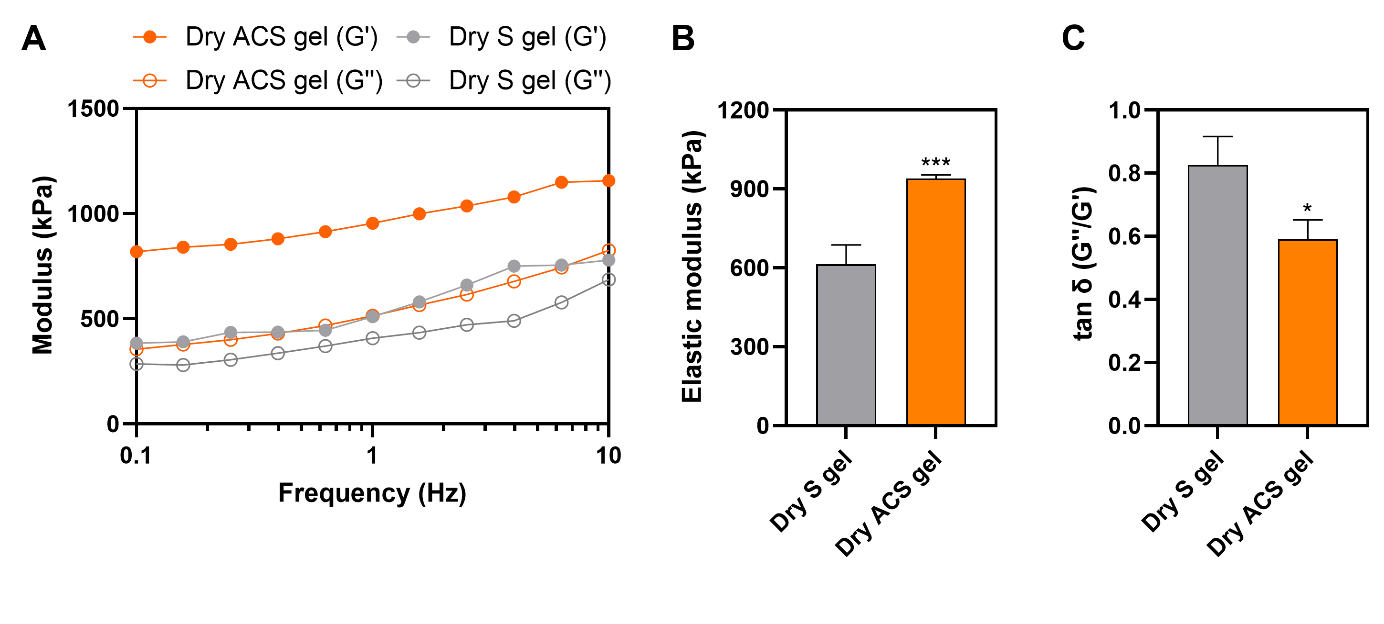


**Figure S2. Mechanical properties of dry starch-based gels.** A) Storage and loss moduli of the dry starch-based gels measured at a frequency range from 0.1 to 10 Hz. B) Average elastic modulus and C) elasticity (tan δ, G″/G′) of the dry gels (*n* = 4; **p* < 0.05, ****p* < 0.001).


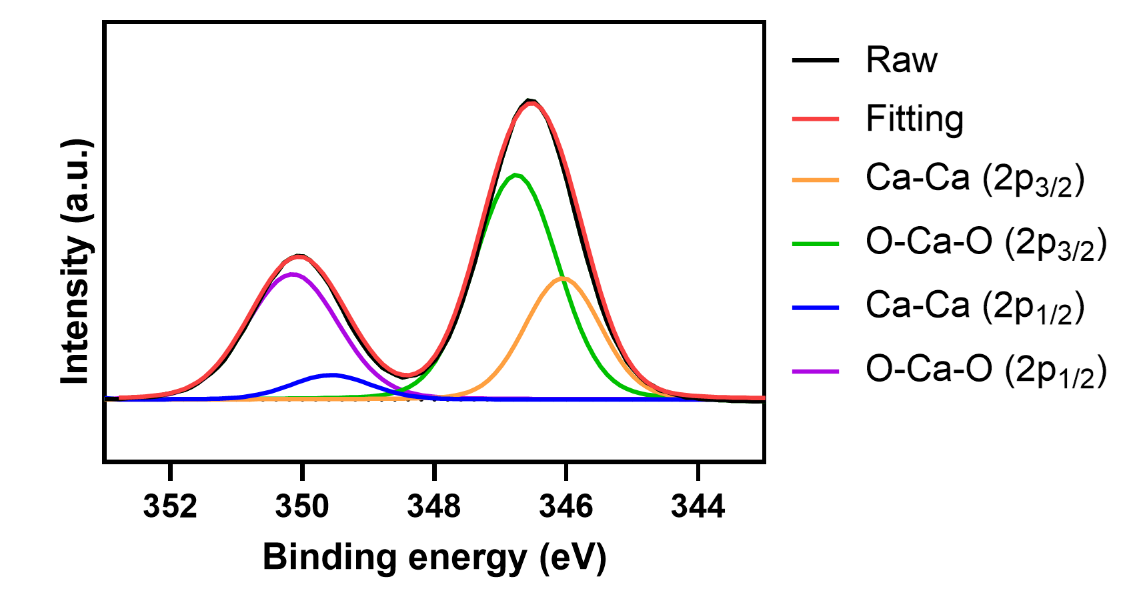


**Figure S3. XPS analysis of calcium-mediated gelation of ACS hydrogel.** Ca2p spectrum deconvoluted into four peaks with binding energy values at 346.1 eV (Ca-Ca bonding, 2p_3/2_), 346.8 eV (O-Ca-O bonding, 2p_3/2_), 349.6 eV (Ca-Ca bonding, 2p_1/2_), and 350.2 eV (O-Ca-O bonding, 2p_1/2_). The ACS gel sample crosslinked with PBS was used for XPS analysis.


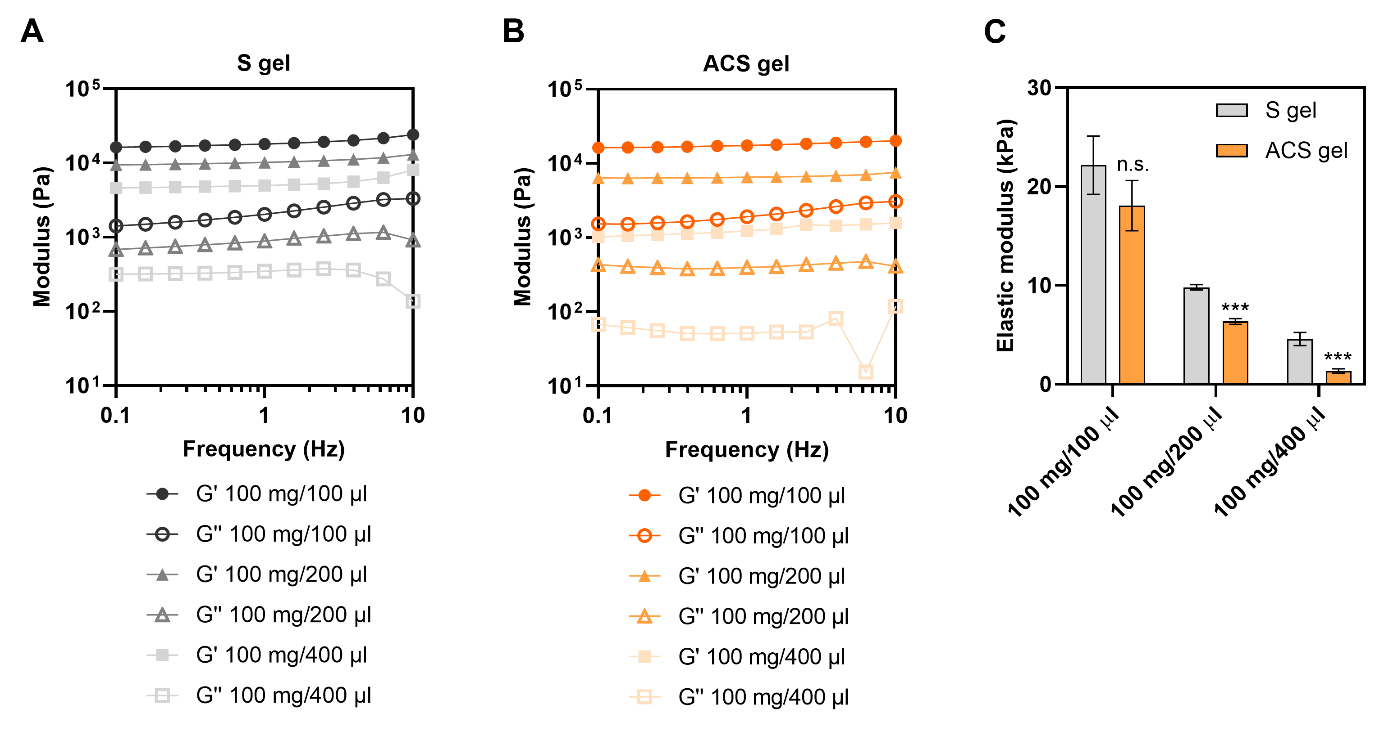


**Figure S4. Mechanical properties of starch-based gels after wetting-mediated gelation of each microparticle (MP).** Storage and loss moduli of A) S gels and B) ACS gels made with the different combinations of MPs and BSA solution (100 mg of particle with 100 μl of solution, 100 mg of particle with 200 μl of solution, and 100 mg of particle with 400 μl of solution). The modulus of each gel was measured at a frequency range from 0.1 to 10 Hz. C) Average elastic modulus of the S gels and ACS gels made with the different combinations of MPs and BSA solution (*n* = 4; ****p* < 0.001, n.s. indicates not statistically significant).


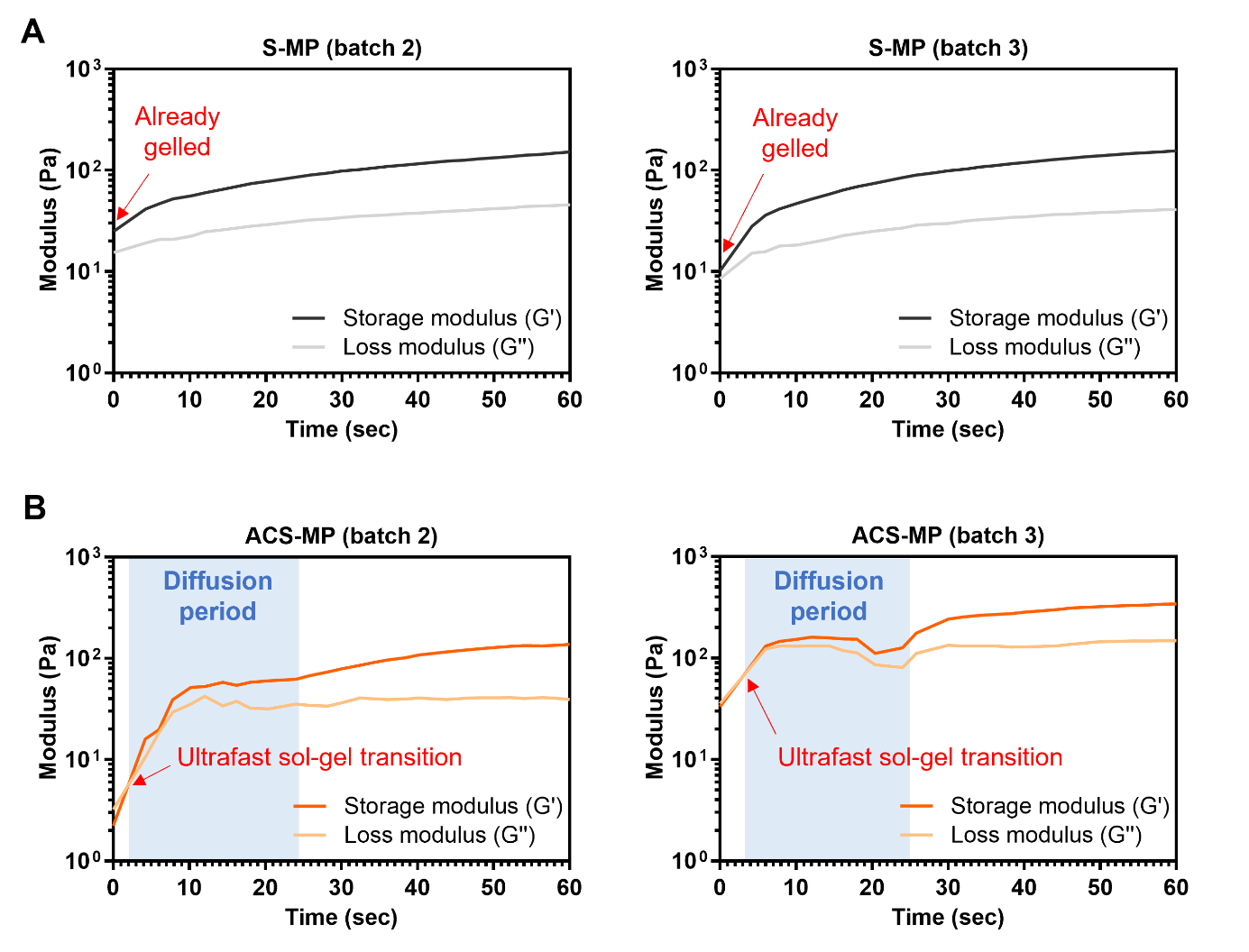


**Figure S5. Gelation kinetics of starch-based microparticles (MPs) from different batches.** Time-dependent changes in the storage and loss moduli of A) S microparticle (S-MP) and B) ACS microparticle (ACS-MP) upon absorbing BSA solution. Different batches of MPs were tested for rheological analysis to investigate gelation kinetics.


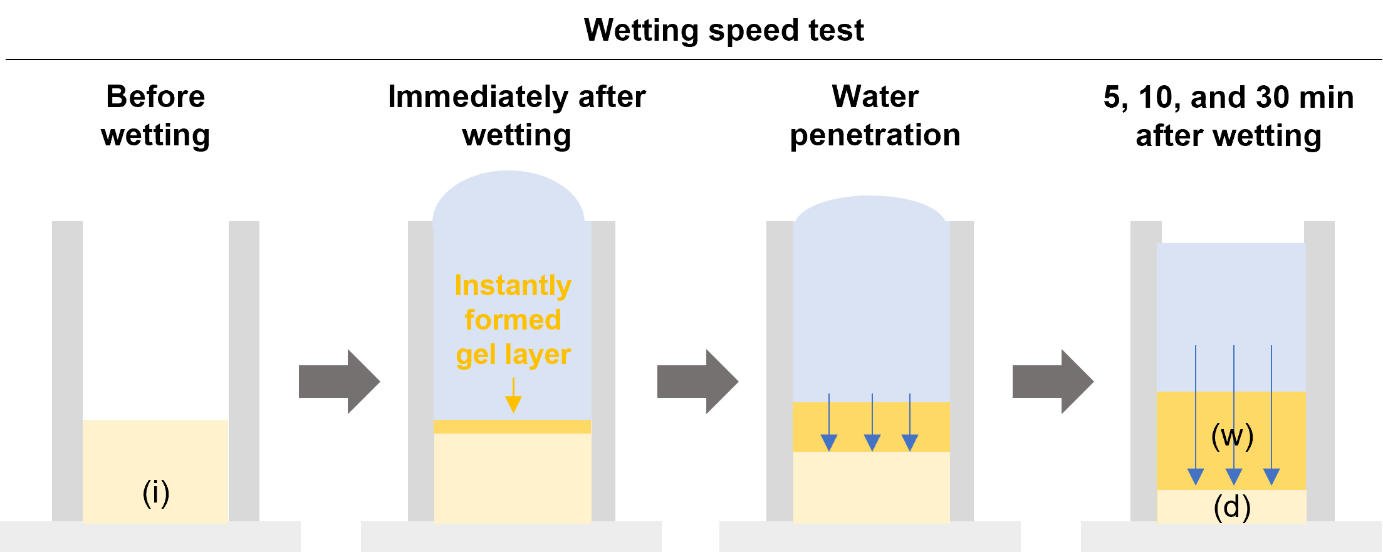


**Figure S6. Schematic illustration of the experimental setting for measuring the wetting speed of starch-based microparticles (MPs).** In this schematics, (i), (d), and (w) indicate the initial MPs, the remaining dry MPs, and the wet MPs, respectively. Detailed method for calculating the wet amount and wetting speed of the MPs is described in the experimental section.


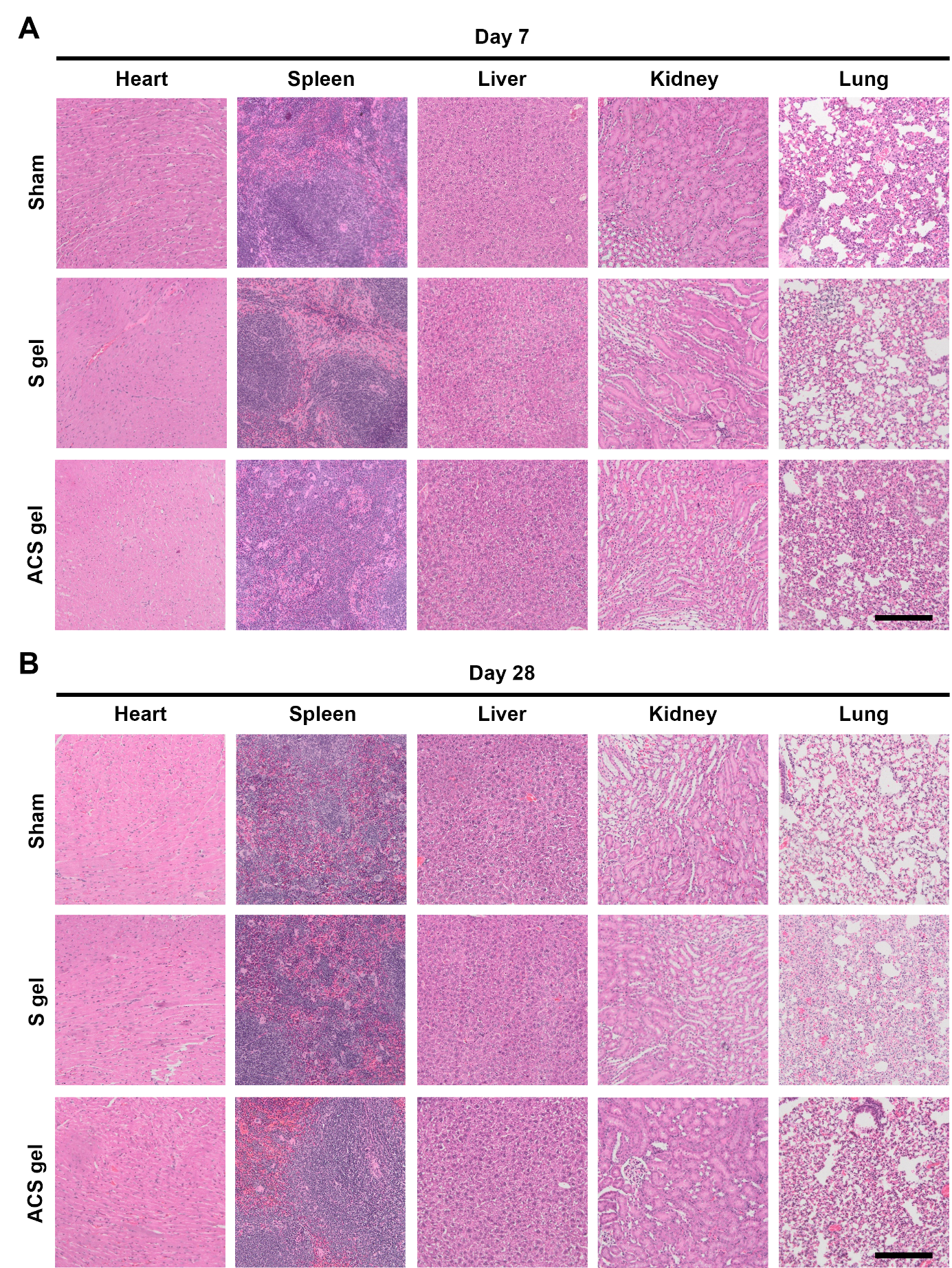


**Figure S7. Histological analysis for checking *in vivo* biocompatibility of starch-based gels.** H&E-stained images of the major organs (heart, spleen, liver, kidney, and lung) harvested from the mice A) 7 days and B) 28 days after subcutaneous implantation of the S gel and ACS gel (scale bars = 200 μm). The mice without gel implantation were used as a sham group.


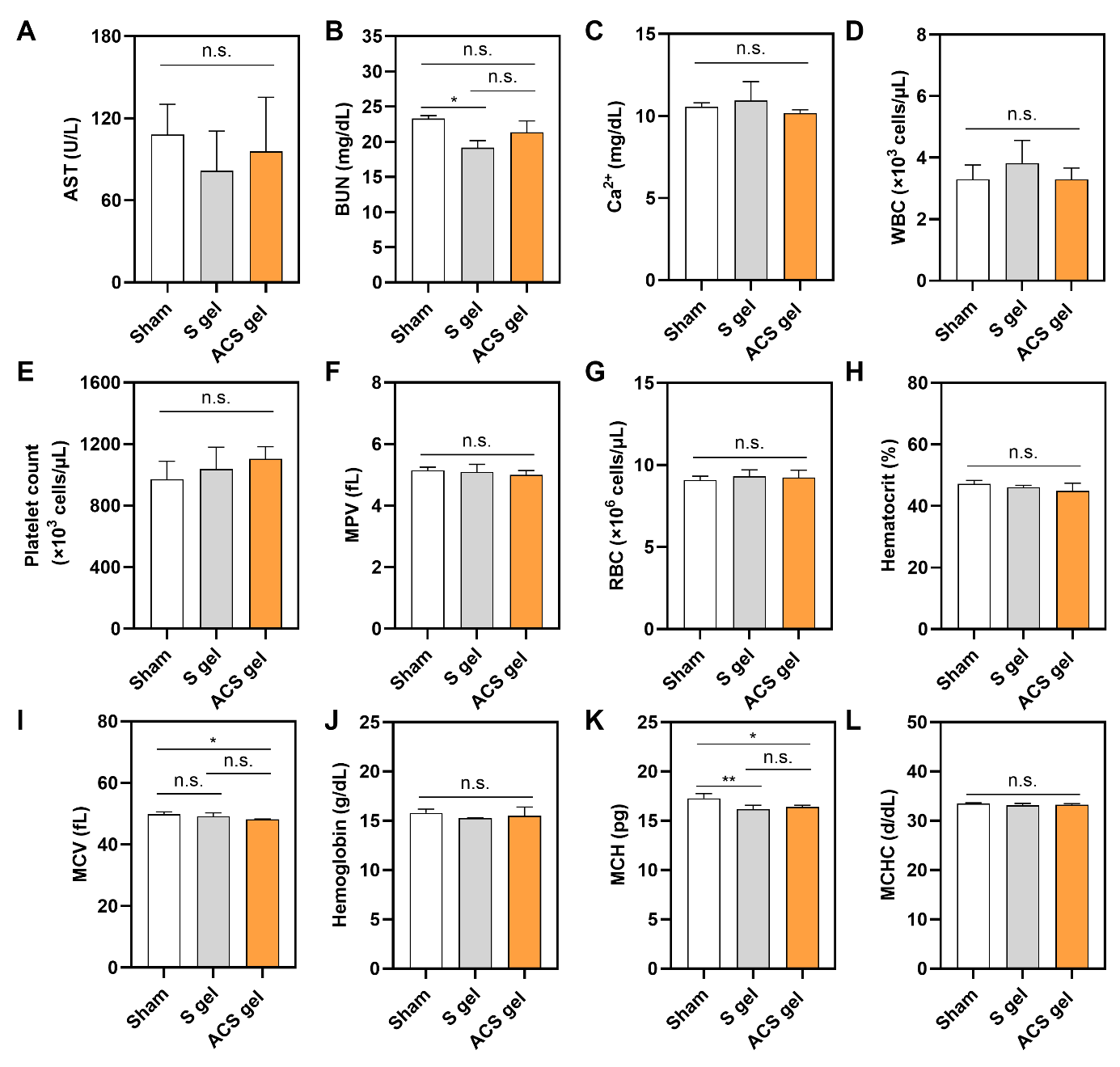


**Figure S8. Blood biochemistry and hematological analysis of the mice seven days after subcutaneous implantation of starch-based gels.** Blood chemistry analysis for quantification of A) aspartate transferase (AST), B) blood urea nitrogen (BUN), and C) calcium ion (Ca^2+^) in the blood collected from the mice in the sham, S gel, and ACS gel groups (*n* = 4; **p* < 0.05). Hematological analysis for quantification of D) white blood cell (WBC), E, F) platelet-related parameters (MPV: mean platelet volume), G–I) red blood cell-related parameters (RBC: red blood cell, MCV: mean corpuscular volume), and J–L) hemoglobin-related parameters (MCH: mean corpuscular hemoglobin, MCHC: mean corpuscular hemoglobin concentration) in the blood collected from the mice in the sham, S gel, and ACS gel groups (*n* = 4; **p* < 0.05, ***p* < 0.01, n.s. indicates not statistically significant).


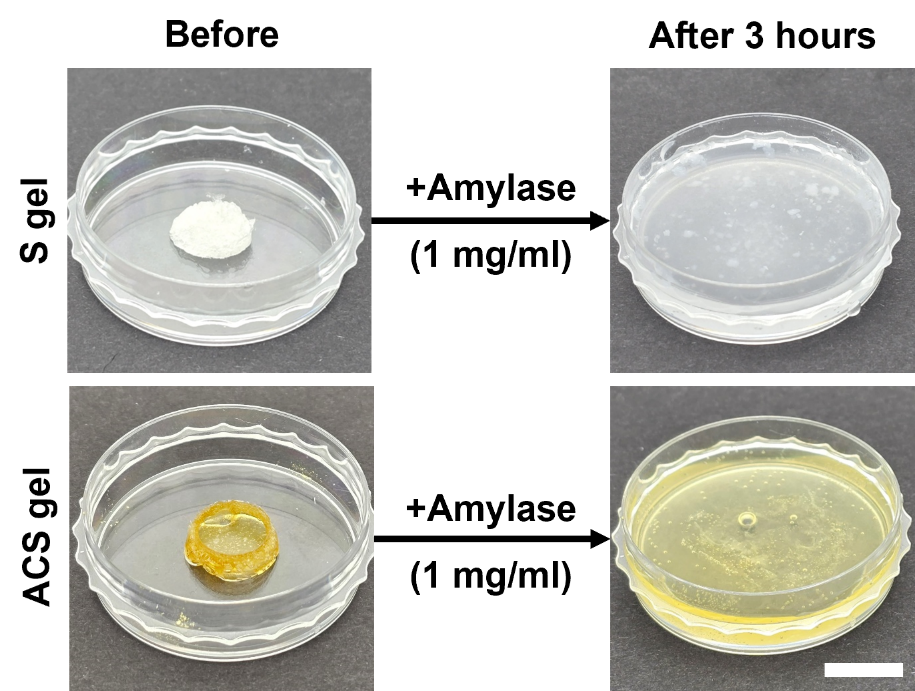


**Figure S9. Biodegradability of starch-based gels.** Photographs of S gel and ACS gel before and 3 hours after treatment of amylase solution (1 mg/ml in PBS) (scale bar = 1 cm).


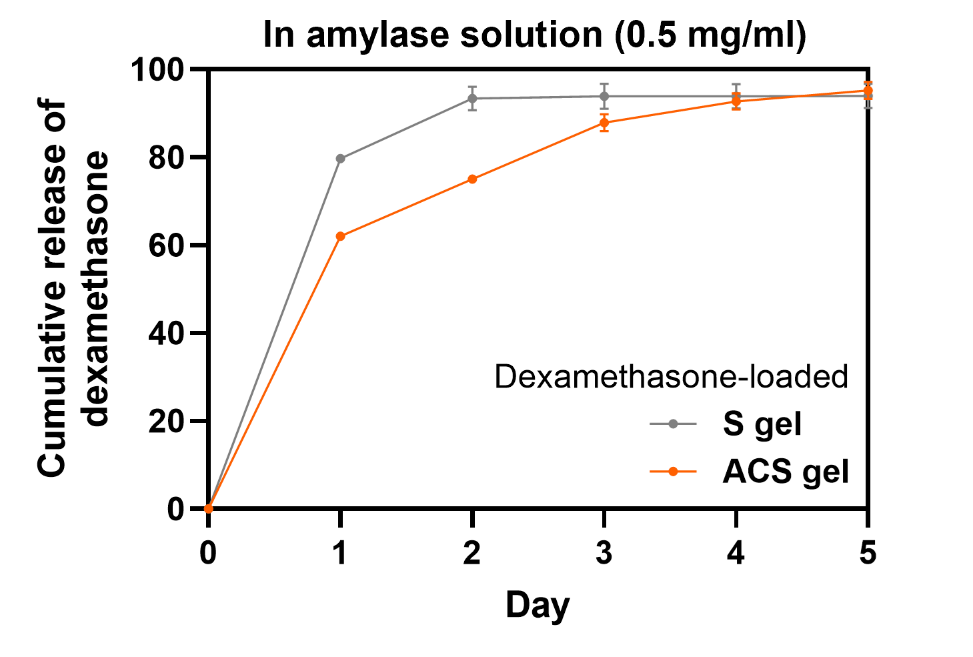


**Figure S10. The potential of starch-based gels as a drug delivery system.** *In vitro* cumulative release profiles of dexamethasone from the drug-loaded S gel and ACS gel during incubation in amylase solution (0.5 mg/ml in PBS) at 37ºC (*n* = 4).

**Table S1. The atomic composition and degree of catechol substitution (DS) in different batches of ACS polymer quantified by XPS analysis.**

| Batch #1 | | | Batch #2 | | | Batch #3 | | |
| --- | --- | --- | --- | --- | --- | --- | --- | --- |
|  | Atomic % | Calculated  DS (%) |  | Atomic % | Calculated  DS (%) |  | Atomic % | Calculated  DS (%) |
| C1s | 63.11 | **13.19** | C1s | 64.61 | **9.41** | C1s | 68.52 | **9.69** |
| N1s | 1.18 |  | N1s | 0.90 |  | N1s | 0.98 |  |
| O1s | 35.71 |  | O1s | 34.49 |  | O1s | 30.5 |  |
